# Expanding the Scope of 2′-SCF_3_ Modified RNA

**DOI:** 10.1002/chem.201500415

**Published:** 2015-06-12

**Authors:** Lukas Jud, Marija Košutić, Veronika Schwarz, Markus Hartl, Christoph Kreutz, Klaus Bister, Ronald Micura

**Affiliations:** [a] Institute of Organic Chemistry, Center for Molecular Biosciences Innsbruck (CMBI), University of InnsbruckInnrain 80–82, 6020 Innsbruck (Austria); [b] Institute of Biochemistry, Center for Molecular Biosciences Innsbruck (CMBI), University of InnsbruckInnrain 80–82, 6020 Innsbruck (Austria)

**Keywords:** fluorine, NMR spectroscopy, oligonucleotides, phosphoramidites, solid-phase synthesis

## Abstract

The 2′-trifluoromethylthio (2′-SCF_3_) modification endows ribonucleic acids with exceptional properties and has attracted considerable interest as a reporter group for NMR spectroscopic applications. However, only modified pyrimidine nucleosides have been generated so far. Here, the syntheses of 2′-SCF_3_ adenosine and guanosine phosphoramidites of which the latter was obtained in highly efficient manner by an unconventional Boc-protecting group strategy, are reported. RNA solid-phase synthesis provided site-specifically 2′-SCF_3_-modified oligoribonucleotides that were investigated intensively. Their excellent behavior in ^19^F NMR spectroscopic probing of RNA ligand binding was exemplified for a noncovalent small molecule–RNA interaction. Moreover, comparably to the 2′-SCF_3_ pyrimidine nucleosides, the purine counterparts were also found to cause a significant thermodynamic destabilization when located in double helical regions. This property was considered beneficial for siRNA design under the aspect to minimize off-target effects and their performance in silencing of the *BASP1* gene was demonstrated.

## Introduction

Chemical modification can significantly enrich the structural and functional repertoire of ribonucleic acids and equip them with new fascinating properties.[1–9] Recently, we have reported the original synthesis of 2′-SCF_3_-modified RNA.[10,11] This modification has considerable potential for broad NMR spectroscopic applications in the nucleic acids field, particularly for probing of RNA–ligand interactions and for monitoring structural rearrangements, both at the secondary and tertiary structure level.[12–21] The main reason for this alluring prospect originates from the very high sensitivity compared to the commonly used single-fluorine labeling patterns that involve mostly 5-fluoro or 2′-fluoro uridines.[13] Surprisingly, we also found that the 2′-SCF_3_ modification has a significant effect on base-pairing strength when positioned in double helical regions, rendering 2′-SCF_3_ nucleosides to one of the most destabilizing 2′-modifications known to date.[11,22] Thereby, the extent of thermodynamic destabilization is comparable to that of nucleic acids containing acyclic (“unlocked”) nucleosides.[23] We have previously investigated this effect which is probably due to the strong preference for C2′-*endo* conformation of the 2′-SCF_3_ ribose moiety.[11] Nevertheless, all our knowledge stems from 2′-SCF_3_ uridine and/or 2′-SCF_3_ cytidine containing RNA exclusively.[10,11] In view of the broad spectrum of applications in chemical biology, we synthesized the novel 2′-SCF_3_ adenosine and 2′-SCF_3_ guanosine phoshoramidites, incorporated them into oligoribonucleotides, studied their physicochemical properties and demonstrated their principal potential for siRNA technologies; all of these features are reported in this article.

## Results and Discussion

### Synthesis of 2′-SCF_3_ adenosine phosphoramidite (A9)

Our synthetic route began with the simultaneous protection of the 5′ and 3′-hydroxyl groups of commercially available 9-(β-d-arabinofuranosyl)adenine using 1,3-dichloro-1,1,3,3-tetraisopropyl disiloxane (TIPDSCl_2_) to furnish the nucleoside intermediate **A1** (Scheme 1),[24] followed by protection of the exocyclic adenine 6-amino group using *N*,*N*-dibutylformamide dimethyl acetal to yield derivative **A2**. After triflation of the arabinose 2′-OH, compound **A3** was treated with potassium thioacetate and 18-crown-6-ether, producing derivative **A4**. Selective cleavage of the acetyl group under basic conditions gave the precursor thiol **A5** in 75 % yield. Only minor amounts (<10 %) of disulfide bridged dimer was observed as a byproduct. The key step, regioselective trifluoromethylation of the thiol group, was achieved in excellent yields using 3,3-dimethyl-1-(trifluoromethyl)-1,2-benziodoxole (Togni’s reagent).[25] Deprotection of the TIPDS moiety of **A6** proceeded in a straightforward manner using tetrabutylammonium fluoride (TBAF) and acetic acid. Finally, compound **A7** was transformed into the dimethoxytritylated derivative **A8**, and conversion into the corresponding phosphoramidite **A9** was achieved in good yields by treatment with 2-cyanoethyl *N*,*N*-diisopropylchlorophosphoramidite. Starting with arabinoadenosine, our route provides **A9** in 26 % overall yield in nine steps with seven chromatographic purifications; in total, 0.5 g of **A9** was obtained in the course of this study.

**Scheme 2:**
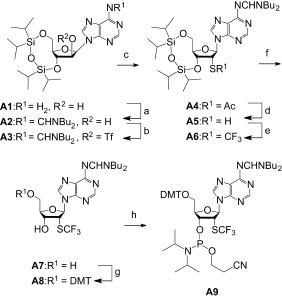
Synthesis of 2′-SCF_3_ adenosine phosphoramidite A9. Reaction conditions: a) 3.0 equiv *N*,*N*-di-*n*-butylformamide dimethyl acetal, in DMF, RT, 26 h, 96 %; b) 1.5 equiv F_3_CSO_2_Cl, 3.0 equiv DMAP, in CH_2_Cl_2_, 0 °C, 20 min; c) 1.5 equiv CH_3_COS^−^K^+^, 1.5 equiv 18-crown-6, in toluene, 17 h, 45 °C, 93 % (over two steps); d) 0.1 M NaOH, in EtOH/pyridine/H_2_O (20:20:1), 0 °C, 10 min, 75 %; e) 1.2 equiv 3,3-dimethyl-1-(trifluoromethyl)-1,2-benziodoxole, in CH_2_Cl_2_, −78 °C to RT, 16 h, 85 %; f) 1 M TBAF, 0.5 M CH_3_COOH, in THF, RT, 1.0 h, 72 %; g) 1.1 equiv DMT-Cl, 0.1 equiv DMAP, in pyridine, RT, 14 h, 87 %; h) 1.5 equiv 2-cyanoethyl *N*,*N*-diisopropylchlorophosphoramidite, 10 equiv CH_3_CH_2_N(CH_3_)_2_, CH_2_Cl_2_, RT, 3 h, 73 %.

### Synthesis of 2′-SCF_3_ guanosine phosphoramidite (G7)

The synthesis of 2′-modified guanosine derivatives usually requires protection of the guanine lactam moiety against electrophilic reagents which is often accomplished by the *O*^6^-(4-nitrophenyl)ethyl group that is introduced under Mitsunobu conditions.[26,27] Although continuously optimized over the years in our laboratory, we experienced unsatisfying yields for this transformation. Additionally, extensive purification protocols were required rendering such a pathway not very attractive. In the present case, we therefore commenced with the *O*^6^-*tert*-butyl, *N*^2^(bis-[*tert*-butyloxycarbonyl]) (*O*^6^-*t*Bu, *N*^2^-Boc_2_) protected 9-(β-d-arabinofuranosyl)guanine derivative **G1** (Scheme 2). This compound is readily available in large quantities from 9-(β-d-arabinofuranosyl)guanine, in analogy to a recent report on the synthesis of guanosine-based amphiphiles.[28–30]

**Scheme 2:**
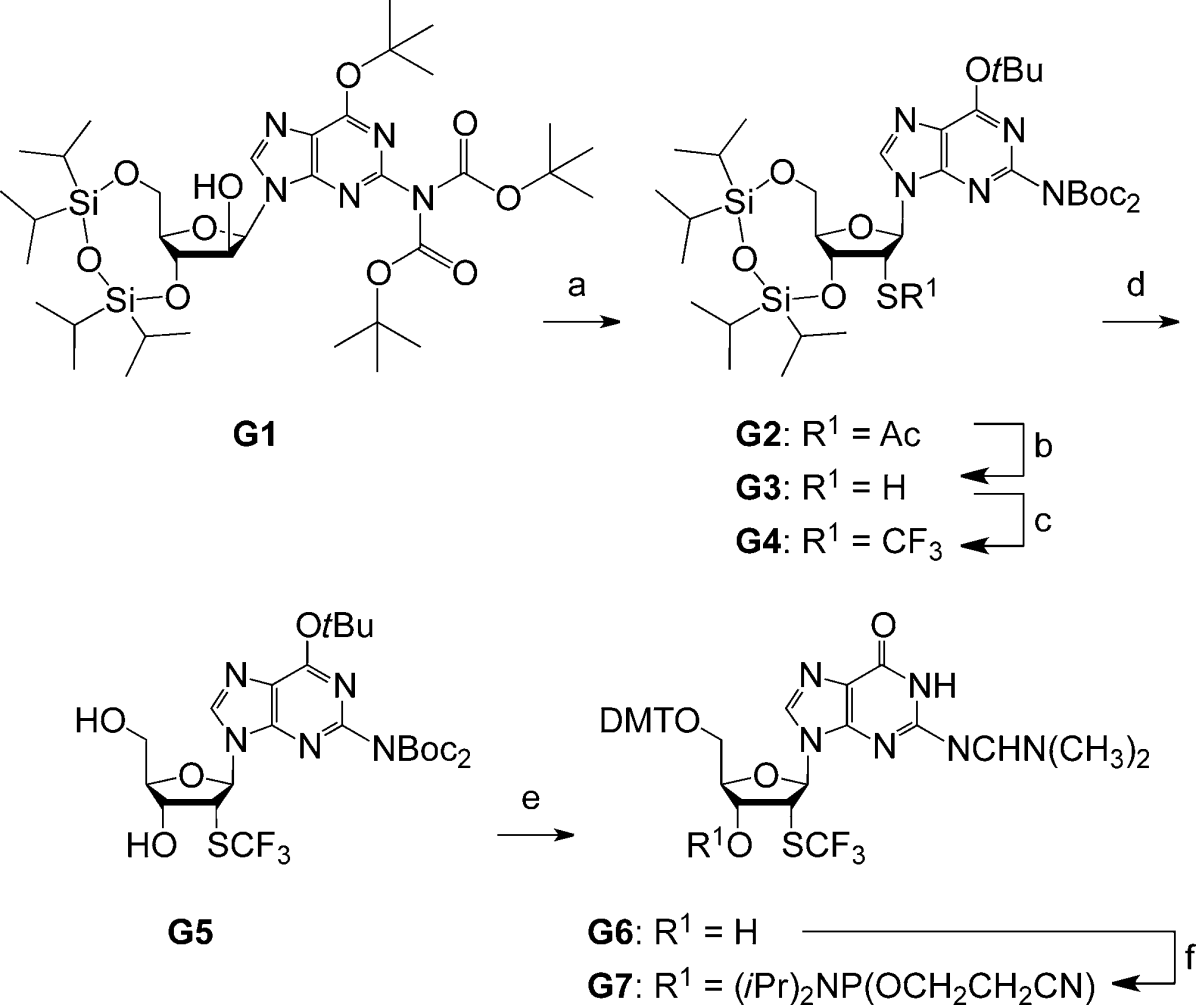
Synthesis of 2′-SCF_3_ guanosine phosphoramidite G7. Starting compound G1 was obtained according to ref. [27]. Reaction conditions: a) i. 1.5 equiv F_3_CSO_2_Cl, 3.0 equiv DMAP, in CH_2_Cl_2_, 0 °C, 20 min; ii. 1.5 equiv CH_3_COS^−^K^+^, 1.5 equiv 18-crown-6, 1.5 equiv EtN(*i*Pr)_2_ in toluene, 16 h, 45 °C, 82 %; b) 1.6 M MeNH_2_, in EtOH/CH_2_Cl_2_ (23:1), 0 °C, 25 min, 85 %; c) 1.2 equiv 3,3-dimethyl-1-(trifluoromethyl)-1,2-benziodoxole, CH_2_Cl_2_, −78 °C to RT, 16 h, 73 %; d) 1 M TBAF, in THF, RT, 3.5 h, 81 %; e) i. CF_3_COOH/CH_2_Cl_2_ (1:7), RT, 2.5 h; ii. 3.0 equiv (H_3_CO)_2_CHN(CH_3_)_2_, in CH_3_OH, reflux, 6 h; iii. 1.1 equiv DMT-Cl, 0.1 equiv DMAP, in pyridine, RT, 18 h, 48 %; f) 1.5 equiv 2-cyanoethyl *N*,*N*-diisopropylchlorophosphoramidite, 10 equiv CH_3_CH_2_N(CH_3_)_2_, CH_2_Cl_2_, RT, 3 h, 72 %.

Therefore, compound **G1** was triflated at the arabinose 2′-OH and subsequently treated with potassium thioacetate and 18-crown-6-ether, producing derivative **G2**. Selective cleavage of the acetyl group under basic conditions gave the precursor thiol **G3** in 85 % yield. Then, regioselective trifluoromethylation of the thiol group was achieved using 3,3-dimethyl-1-(trifluoromethyl)-1,2-benziodoxole (Togni’s reagent)[25] and provided derivative **G4** in high yields. Deprotection of its TIPDS moiety was straightforward using tetrabutylammonium fluoride (TBAF). Then, a series of transformations starting with *t*Bu and Boc deprotection of nucleoside **G5** using trifluoro acetic acid, followed by amidine protection of the exocyclic amino group and dimethoxytritylation of the 5′-OH group, were optimized in a one-pot procedure requiring only a single chromatographic purification of the target compound **G6**. Conversion into the corresponding phosphoramidite was achieved in good yields by treatment with 2-cyanoethyl *N*,*N*-diisopropylchlorophosphoramidite. Our route provides **G7** in a 14 % overall yield in six steps with six chromatographic purifications; in total, 0.7 g of **G7** was obtained in the course of this study.

We point out that the Boc protection concept proved very convenient and was the key for the high efficiency of the synthesis. Therefore, we currently plan to integrate the Boc approach for the syntheses of other 2′-modified (e.g., 2′-SeCH_3_ or 2′-N_3_)[26,27] guanosine building blocks as well.

### Synthesis of 2′-SCF_3_-containing RNA

**Figure 1:**
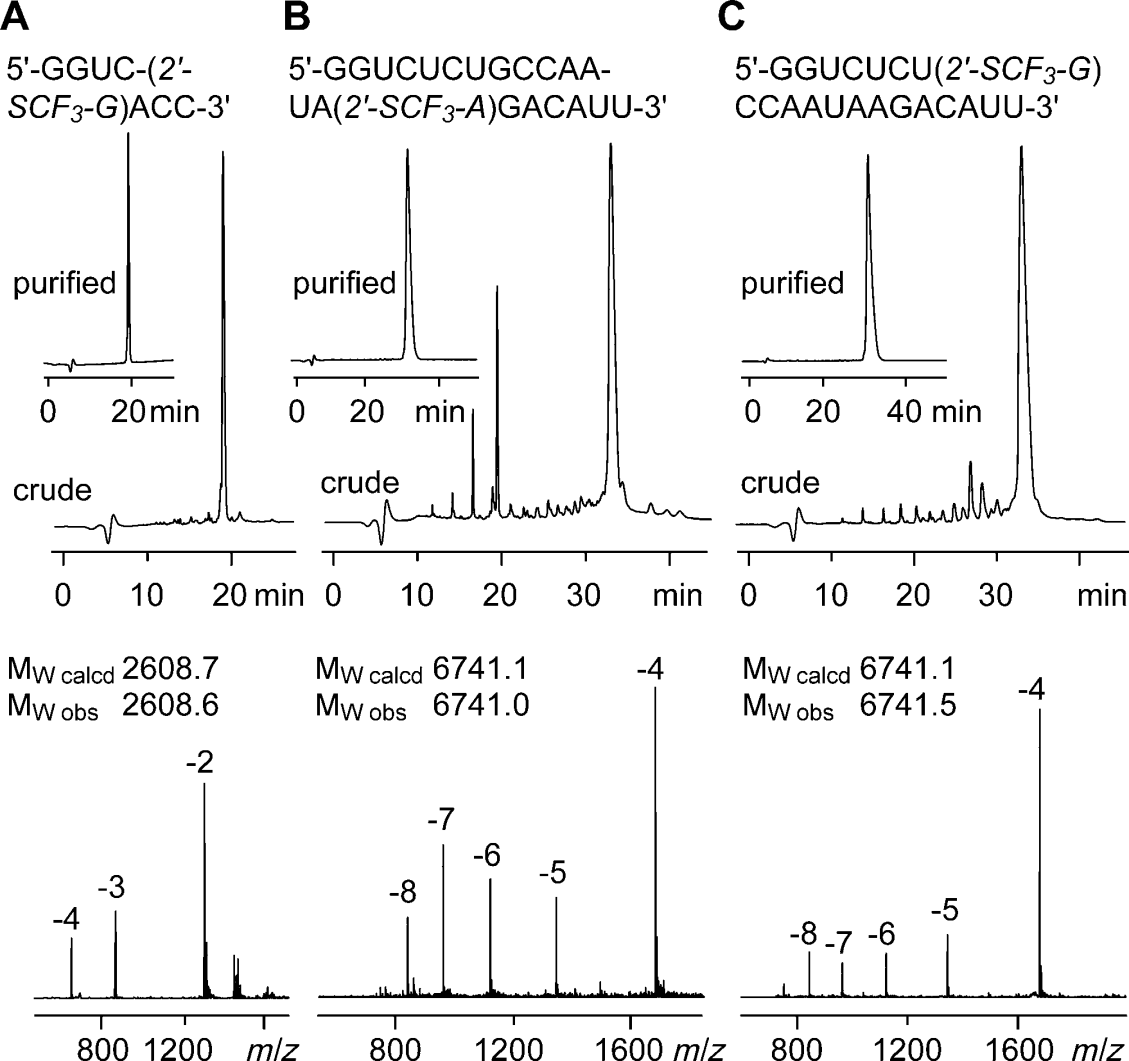
Characterization of 2′-SCF_3_ modified RNA. Anion-exchange HPLC traces (top) of: A) 8 nt RNA, B) 21 nt RNA, and C) 21 nt RNA, and the corresponding LC-ESI mass spectra (bottom). HPLC conditions: Dionex DNAPac column (4×250 mm), 80 °C, 1 mL min^−1^, 0–60 % buffer B in 45 min; buffer A: Tris-HCl (25 mM), urea (6 M), pH 8.0; buffer B: Tris-HCl (25 mM), urea (6 M), NaClO_4_ (0.5 M), pH 8.0. For LC-ESI MS conditions, see the Supporting Information.

We used the 2′-*O*-TOM approach for the solid-phase synthesis of RNA with site-specific 2′-SCF_3_ adenosine and guanosine modifications.[31,32] Coupling yields of the two novel building blocks were higher than 98 % according to the trityl assay. The oligoribonucleotides were cleaved from the solid support and deprotected using CH_3_NH_2_ in ethanol/H_2_O, followed by treatment with tetrabutylammonium fluoride (TBAF) in tetrahydrofuran (THF). Subsequent size-exclusion chromatography on a Sephadex G25 column removed salts. The RNA sequences were purified by anion-exchange chromatography under strong denaturating conditions (6 M urea, 80 °C). The molecular weights of the purified RNAs were confirmed by liquid chromatography (LC) electrospray ionization (ESI) mass spectrometry (MS). A selection of 2′-SCF_3_ RNA sequences is listed in the Supporting Information, Table S1. Noteworthy, 2′-SCF_3_ guanosine (such as the previously investigated 2′-SCF_3_ uridine and 2′-SCF_3_ cytidine)[10,11] appeared completely stable under the repetitive oxidative conditions (20 mM aqueous iodine solution) required during RNA solid-phase synthesis for the transformation of P^III^ into P^V^, and the subsequent deprotection (Figure 1). Unexpectedly, the 2′-SCF_3_ adenosine label turned out to be sensitive during the standard deprotection procedure. Only when we added millimolar amounts of *threo*-1,4-dimercapto-2,3-butandiol (DTT), a high quality of the crude deprotected RNA was achieved as analyzed from the corresponding ion-exchange HPLC traces (Figure 1 B) and mass spectrometric experiments. We hypothesize that possible oxidation products (such as sulfoxides, 2′-SOCF_3_) of the protected RNA were reduced by this additive, and hence, follow-up side-products that otherwise dominated during RNA deprotection at high pH values (as a result of sulfoxide elimination and successive strand cleavage) could not form any more. This observation is reminiscent of the chemical synthesis of 2′-SeCH_3_ RNA that we investigated several years ago;[33] for 2′-SeCH_3_ guanosine-modified RNA, the corresponding oxidation products were analyzed in detail by mass spectrometry, and additionally isolated and quantitatively reduced by DTT.[26] Unfortunately, our attempts to isolate oxidized species of 2′-SCF_3_ RNA have failed so far.1

### Base pairing properties of 2′-SCF_3_-containing RNA

**Figure 2:**
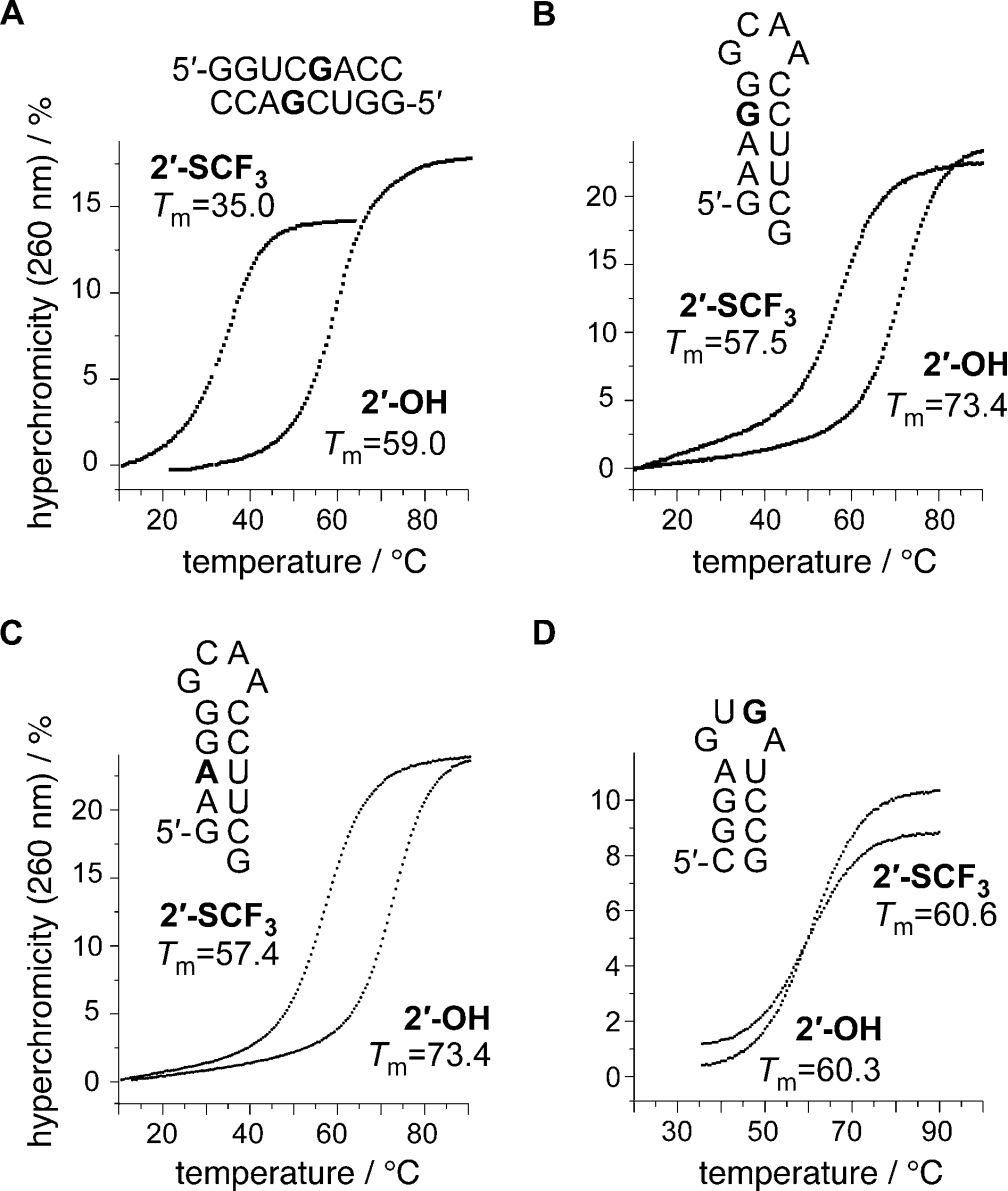
Thermal stabilities of unmodified and 2′-SCF_3_ modified oligoribonucleotides. UV-melting profiles of A) self-complementary 8 nt RNA, B) 15 nt RNA hairpin, C) 15 nt RNA hairpin, and D) 12 nt RNA hairpin. Conditions: *c*_RNA_=8 μM; 10 mM Na_2_HPO_4_, 150 mM NaCl, pH 7.0. Nucleotide abbreviations in bold indicate the positions for 2′-SCF_3_ modification.

A single 2′-SCF_3_ adenosine or 2′-SCF_3_ guanosine exhibited a pronounced attenuation of RNA duplex stability provided that the modification was located in the Watson–Crick base-pairing region. For instance, UV melting profile analysis of the palindromic RNA 5′-GGUC(2′-SCF_3_-G)ACC (Figure 2 A) revealed an average decrease of 24 °C in *T*_m_ values for RNA concentrations in the micromolar range (Δ*G*°, −7.8 kcal mol^−1^; Δ*H*°, −72.3 kcal mol^−1^; Δ*S*°, −216 cal mol^−1^ K^−1^), compared to the unmodified counterpart (Δ*G*°, −15.4 kcal mol^−1^; Δ*H*°, −84.8 kcal mol^−1^; Δ*S*°, −233 cal mol^−1^ K^−1^). As a second example, the hairpin-forming RNA 5′-GAA(2′-SCF_3_-G)G-GCAA-CCUUCG (Figure 2 B) also showed a decrease (14 °C) of *T*_m_ values determined at micromolar RNA concentrations (Δ*G*°, −5.3 kcal mol^−1^; Δ*H*°, −54.7 kcal mol^−1^; Δ*S*°, −166 cal mol^−1^ K^−1^), compared to the unmodified counterpart (Δ*G*°, −7.1 kcal mol^−1^; Δ*H*°, −52.1 kcal mol^−1^; Δ*S*°, −151 cal mol^−1^ K^−1^). Likewise, the same hairpin sequence but with 2′-SCF_3_ adenosine, 5′-GA(2′-SCF_3_-A)GG-GCAA-CCUUCG (Figure 2 C), was destabilized (Δ*G*°, −5.5 kcal mol^−1^; Δ*H*°, −57.9 kcal mol^−1^; Δ*S*°, −176 cal mol^−1^ K^−1^).2

**Figure 3:**
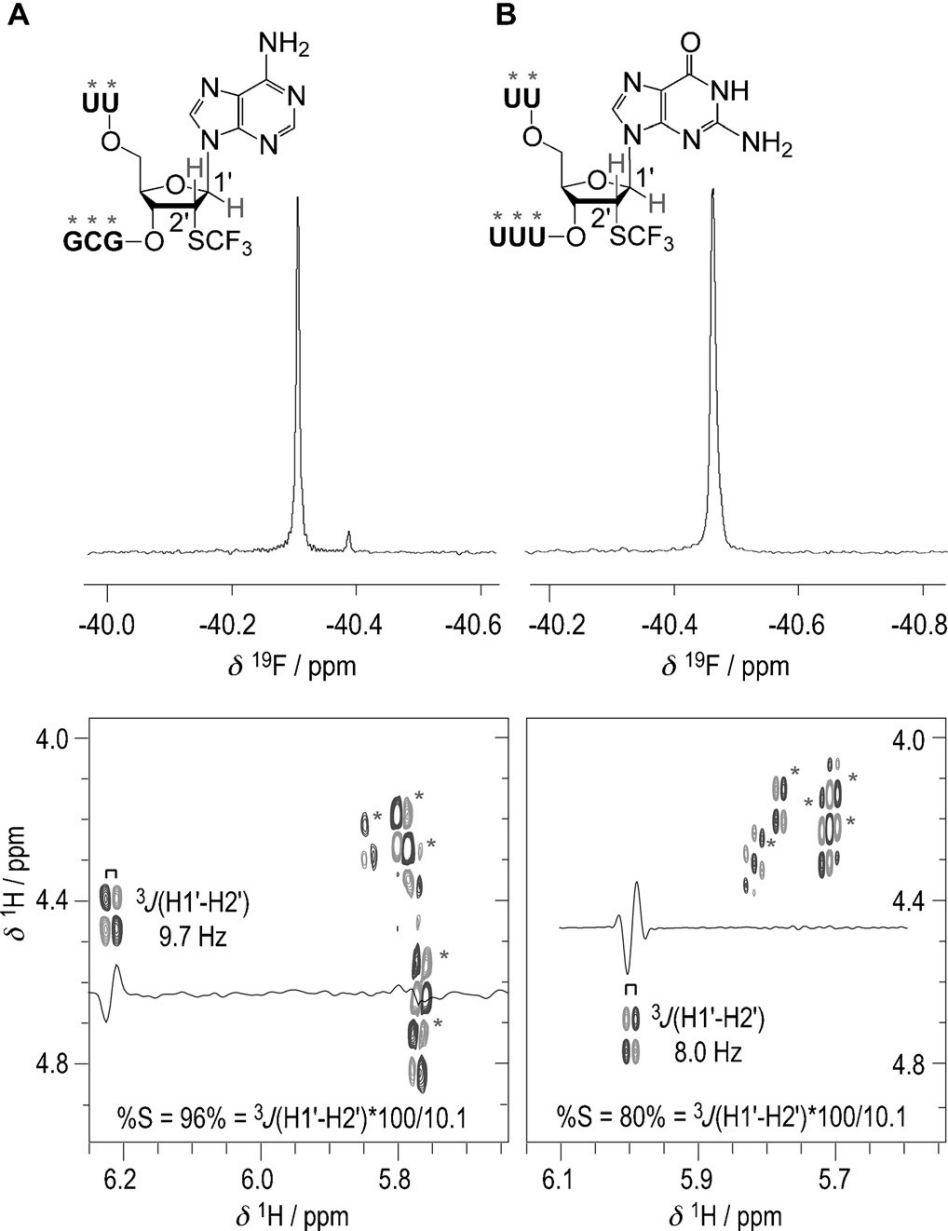
^19^F and ^1^H, ^1^H ECOSY NMR spectra of single-stranded RNAs: A) 5′-UU(2′-SCF_3_-A)GCG, and B) 5′-UU(2′-SCF_3_-G)UUU. For the 2′-SCF_3_ moiety, the 3-bond scalar coupling constants of H1′ and H2′ (^3^*J*H1′-H2′) were determined to be 9.7 and 8.0 Hz, respectively. These account for 96 and 80 % C2′-*endo* (South) populations in C2′/C3′-*endo* equilibria.[35,36] Note that for pyrimidine nucleosides the C2′-*endo* (South) population was 100 %.[10,11] Conditions: *c*_RNA_=0.3 mM; 25 mM sodium cacodylate, pH 7.0, 298 K.

The influence of the 2′-SCF_3_ purine nucleosides on thermodynamic parameters of double helix stability was therefore comparable to the corresponding 2′-SCF_3_ pyrimidine series investigated previously, for which the destabilization was attributed to the inherent preference for C2′-*endo* conformation of these nucleosides.[11] To support such a hypothesis also for the purine nucleoside series investigated here, we synthesized the short, single-stranded RNAs, 5′-UU(2′-SCF_3_-A)GCG, and 5′-UU(2′-SCF_3_-G)UUU, and determined ^3^*J*(H1′-H2′) coupling constants by 2D ^1^H,^1^H exclusive correlation spectroscopy (ECOSY) (Figure 3). For 2′-SCF_3_-adenosine and -guanosine, the values were determined to be 9.7 and 8.0 Hz, respectively, accounting for a population of 96 and 80 % of C2′-*endo* ribose conformation in single-stranded RNA.3

As a consequence, this observation portends that forcing a 2′-SCF_3_ nucleoside into a C3′-*endo* (or C3′-*endo*-like) ribose pucker, as mandatory for an A-form RNA double helix to avoid steric interference of the 2′-substituent, would introduce an energetic penalty. Importantly, a very recent computational study by Li and Szostak supports this hypothesis.[34] The calculated free energy landscape revealed that the C2′-*endo* conformation of a single nucleoside within a native A-form RNA duplex is significantly less stable by 6 kcal mol^−1^ compared to the C3′-*endo* conformer.[34] This large value accounts for the disruption of the planar base-pair structure (therefore weakening stacking and hydrogen-bonding interactions) if a C2′-*endo* ribose had to be accommodated into the overall A-form geometry.[34]

We recall that 2′-SCF_3_ pyrimidine nucleosides exerted only a minor or negligible effect on thermodynamic stability if located in *single*-stranded regions (such as loops, bulges, or overhangs) next to double helixes.[11] This behavior was also found for the corresponding purine nucleosides investigated here. For instance, the hairpin forming RNA 5′-CGGA-GUGA-UCCG (*T*_m_=60.3 °C; Δ*G*°, −5.7 kcal mol^−1^; Δ*H*°, −54.4 kcal mol^−1^; Δ*S*°, −163 cal mol^−1^ K^−1^) showed thermodynamic parameters that were comparable to those of the modified counterpart of 5′-CGGA-GU(2′-SCF_3_-G)A-UCCG (*T*_m_=60.6 °C; Δ*G*°, −5.7 kcal mol^−1^; Δ*H*°, −55.3 kcal mol^−1^; Δ*S*°, −166 cal mol^−1^ K^−1^; Figure 2 D).

In response to a reviewer’s comment, we point out that the 2′-SCF_3_ modification is destabilizing also in the context of a DNA double helix. This is not unexpected because the C2′-*endo* pucker exposes the C2′-substituent in ribose configuration (such as the 2′-SCF_3_ group used here) to steric hindrance with the phosphate backbone in B-form conformation. In preliminary experiments, we found that the destabilization is of comparable degree for DNA and RNA. For example, the DNA hairpin 5′-CCGGAAGGT-ACGA-ACCTTCCG-3′ melts at a *T*_m_ value of 73.8 °C in 10 mM Na_2_HPO_4_, 150 mM NaCl, pH 7.0 (Δ*G*°, −6.8 kcal mol^−1^; Δ*H*°, −49.0 kcal mol^−1^; Δ*S*°, −142 cal mol^−1^ K^−1^) while 5′-CCGGAAGGT-ACGA-ACC*U_SCF3_*TCCG-3′ melts 23 degrees lower under the same conditions (*T*_m_=50.7 °C; Δ*G*°, −3.4 kcal mol^−1^; Δ*H*°, −41.9 kcal mol^−1^; Δ*S*°, −129.2 cal mol^−1^ K^−1^; Supporting Information, Figure S1).

### Probing of RNA structures by ^19^F NMR spectroscopy

**Figure 4:**
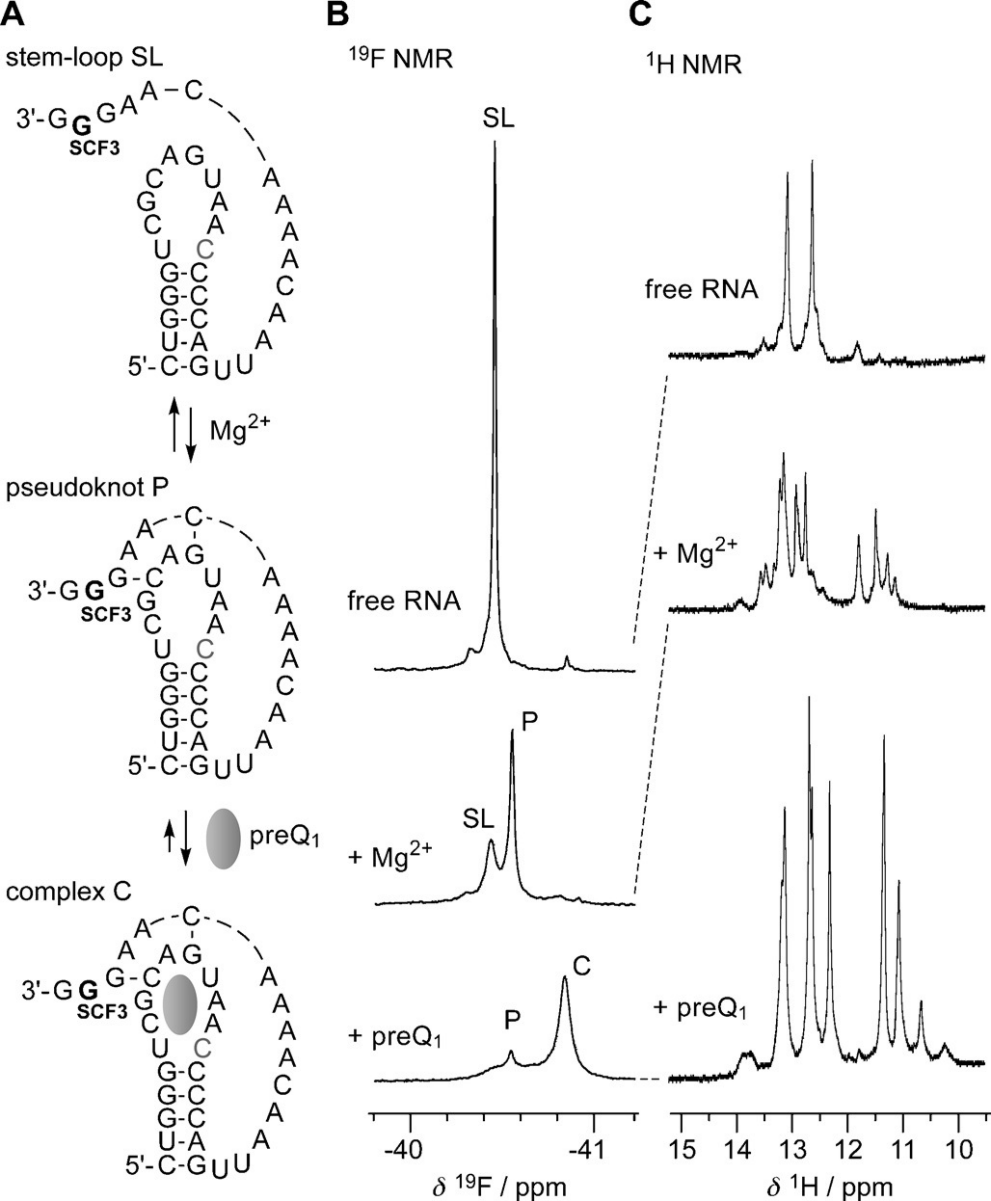
NMR spectroscopic analysis of Mg^2+^-assisted RNA pseudoknot formation, and subsequent stabilization through binding of a small ligand (*Thermoanaerobacter tengcongensis* preQ_1_ class-I riboswitch), using a 2′-SCF_3_ guanosine label. A) RNA secondary structure model, B) corresponding ^19^F NMR spectra, and C) imino proton ^1^H NMR spectra. Conditions: *c*_RNA_=0.3 mM, 25 mM sodium cacodylate, pH 7.0, 298 K; additions: *c*_Mg2+_=2.0 mM; followed by *c*_preQ1_=1.2 mM. The cytosine that forms a Watson–Crick base pair with the preQ_1_ ligand is highlighted in grey.

To evaluate the applicability, and importantly, the uniformity of the 2′-SCF_3_ labeling concept not only with respect to pyrimidine but also with respect to purine nucleosides, we demonstrate a single-case study for NMR spectroscopic RNA probing here. Figure 4 A depicts the secondary structure model for the *Thermoanaerobacter tengcongensis* preQ_1_ class-I riboswitch.[37–39] This RNA becomes preorganized into a pseudoknot fold when Mg^2+^ is present at physiological concentrations. The distribution between the stem-loop fold (SL) with an unpaired strand overhang and the more compact RNA pseudoknot (P) was nicely reflected by the two ^19^F resonances at −40.2 and −40.5 ppm, in 3:7 ratio (Figure 4 B, middle). Ligand addition in fourfold excess resulted in a dominant population of the preQ_1_-bound RNA complex (C), reflected by a new signal at −40.8 ppm (Figure 4 B, bottom). The simplicity of the population analysis based on ^19^F spectra becomes obvious from a direct comparison with the corresponding NH imino proton ^1^H NMR spectra (Figure 4 C). Note that the imino protons exchange with the solvent and therefore result in much weaker intensities for the two dynamic, ligand-unbound RNA conformations (Figure 4 C, top, middle) while signal intensities are higher for the significantly more stable preQ_1_–aptamer complex (Figure 4 C, bottom), hence impairing an accurate quantification of populations of the coexisting folds.4

As a second example for ^19^F NMR spectroscopic applications of the novel labels, we analyzed duplex formation by titration and in temperature-dependent manner. The 14 bp RNA contained a single 2′-SCF_3_ adenosine in the middle region. Its melting temperature was readily obtained. The corresponding set of data is depicted in the Supporting Information, Figure S2.

The NMR analysis presented in Figure 4 together with other examples that we demonstrated previously for the pyrimidine series makes us confident that the 2′-SCF_3_ label awaits rapid and widespread applications.[10,11] Its performance confirms our original expectations for facile NMR spectroscopic probing of RNA structure rearrangements and RNA–ligand interactions.

We also mention that, to the best of our knowledge, only two other CF_3_ sensor nucleosides for probing of RNA secondary structures have been reported so far, namely 4′-C-[(4-trifluoromethyl-1*H*-1,2,3-triazol-1-yl)methyl]thymidine[40] and 5-[4,4,4-trifluoro-3,3-bis(trifluorometh-yl)but-1-ynyl]-2′-deoxyuridine,[41,42] both are sterically more demanding and represent DNA units. Additionally, trifluorothymidine has been analyzed for its NMR spectroscopic properties within DNA.[43]

### RNA interference by 2′-SCF_3_-modified siRNA

As a novel application for the 2′-SCF_3_ modification, we tested the potential of this modification for gene silencing by small interfering RNA (siRNA). Nucleosides with destabilizing effects on Watson–Crick base pairing are of specific interest for the development of oligonucleotide therapeutics.[2,4,23] Most prominent is the highly flexible unlocked nucleic acid (UNA; or “seconucleoside”) modification.[23] UNA, missing the covalent C2′=C3′ bond of a ribose sugar, is not conformationally restrained, and can be used to influence oligonucleotide flexibility. UNA inserts reduce duplex *T*_m_ values by 5 to 10 °C per insert,[23] they facilitate antisense strand selection as the RISC guide, and UNA modifications to the seed region of a siRNA guide strand can significantly reduce off target effects.[44]

The comparable extent of destabilization of UNA and 2′-SCF_3_ modifications prompted us to explore a potential role of the latter in siRNA approaches. For reasons of comparability, we employed the same model system used previously to knock down the brain acid soluble protein 1 (BASP1) encoding gene by transient siRNA nucleofection in the chicken DF-1 cell line.[45,46] Expression of the *BASP1* gene is specifically suppressed by Myc, an evolutionary conserved oncoprotein;[47] conversely, the BASP1 protein is an efficient inhibitor of Myc-induced cell transformation.[46]

**Figure 5:**
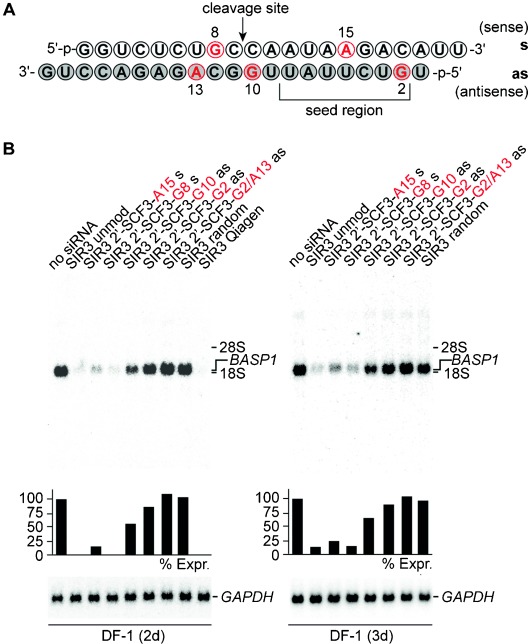
Gene silencing by 2′-SCF_3_-modified siRNAs. A) Sequence of the brain acid soluble protein 1 gene (*BASP1*)[46] targeting siRNA duplex used in this study; nucleosides in red indicate positions for 2′-SCF_3_ modification tested. B) Biological activities of 2′-SCF_3_-adenosine or 2′-SCF_3_-guanosine modified siRNAs directed against *BASP1* mRNA. Chicken DF-1 cells grown on 60 mm dishes were transiently nucleofected with 0.24 nmol (∼3.0 μg) aliquots of unmodified (SIR) or modified siRNAs (SIR 2′-SCF_3_) on sense (s) or antisense (as) strands. An equal aliquot of siRNA with a shuffled (random) nucleotide sequence was used as a control. Total RNAs were isolated 2 days (left panel) or 3 days (right panel) after siRNA delivery, and 5 μg aliquots were analyzed by Northern hybridization using a DNA probe specific for the chicken *BASP1* gene, and subsequently a probe specific for the housekeeping quail *GAPDH* gene.[46] The levels [%] of *BASP1* expression (Expr.) were determined using a phosphorimager and are depicted as bars relative to mock transfections (no SIR, 100 %). The electrophoretic positions of rRNAs are indicated in the margin. SIR random: 5′-UCUGGGUCUAAGCCAAACAUT/5′-UGUUUGGCUUAGACCCAGAUdG.

We synthesized five siRNA duplexes for the *BASP1* target gene with the sequence organization depicted in Figure 5 A (see also the Supporting Information, Table S2), two of them with a single 2′-SCF_3_ adenosine (A15) or guanosine (G8) in the sense strand, two of them with a single 2′-SCF_3_ guanosine modification in the antisense strand, very close to (G10) or within (G2) the seed region, and another one with two 2′-SCF_3_ modifications (G2 and A13) in the antisense strand. We determined the thermodynamic parameters for two of the five modified siRNAs (A15 s and G2 as; seed region) by UV melting profile analysis and—as expected—found significant destabilization compared to the native siRNA (Supporting Information, Figure S3).

The modified siRNAs caused complete gene silencing as observed for the non-modified reference duplex only when the modification resided in the sense strand (Figure 5 B). Instead, siRNA activity was impaired for the 2′-SCF_3_–G10 antisense-modified siRNA. Not unexpectedly, the two siRNAs carrying the 2′-SCF_3_ modification in the seed region at the critical position 2 of the antisense strand[23] were not active. These results indicate that the 2′-SCF_3_ modification is a promising tool for the alternative design of siRNAs with reduced off-target effects and warrants comprehensive studies in the future along these lines.[44]

## Conclusion

The ribose 2′-trifluoromethylthio group makes ribonucleic acids an attractive reporter for spectroscopic investigations of RNA structure, structural dynamics, folding, and ligand interactions. So far, only RNA with 2′-SCF_3_ modified pyrimidine nucleosides has been accessible by chemical synthesis. The syntheses of the novel 2′-SCF_3_ purine nucleoside phosphoramidites and the corresponding RNAs has been demonstrated in this work and significantly expands the scope of applications for this modification. Their excellent behavior in ^19^F NMR probing of structure preformation and ligand binding was exemplified for the preQ_1_ class-I riboswitch and for melting of an RNA duplex. Moreover, all 2′-SCF_3_-modified nucleosides cause thermodynamic destabilization when they reside in double helices. Since this property is reminiscent of “unlocked nucleic acid” (UNA) which is widely used for siRNA technologies to minimize off-target effects,[23,44] we have highlighted the principal potential of 2′-SCF_3_ RNAs for siRNA design as a promising novel application of this modification.

## Experimental Section

### *O^6^-tert*-Butyl-*N,N*-bis(*tert*-butyloxycarbonyl)-3′,5′-*O*-(1,1,3,3-tetraisopropylsiloxane-1,3-diyl)-2′-acetylthio-2′-deoxyguanosine (G2)

Nucleoside **G1** (1.46 g, 1.86 mmol) was dissolved in dichloromethane (25 mL) and DMAP (696 mg, 5.70 mmol) and trifluoromethanesulfonyl chloride (298 μL, 2.80 mmol) was added at 0 °C. After 40 min, the reaction mixture was treated with aqueous NaHCO_3_ solution (5 %), the organic layer was separated, dried over Na_2_SO_4_ and evaporated to yield the 2′-triflated derivative of **G1** as a dark-yellow foam under high vacuum. The residue was dissolved in DMF (25 mL) and treated with potassium thioacetate (319 mg, 2.79 mmol), overnight, at ambient temperature. The solvent was distilled under reduced pressure and the crude product was purified by column chromatography on SiO_2_ (0–4 % CH_3_OH in dichloromethane v/v) to yield **G2** as a brown foam (1.28 g, 1.52 mmol, 82 % over two steps). TLC (3 % CH_3_OH in CH_2_Cl_2_) *R*_f_=0.72. ^1^H NMR (300 MHz, CDCl_3_): *δ=*1.06 (m, 28 H, 2×((CH_3_)_2_CH)_2_Si), 1.39 (s, 18 H, C(2)-N(Boc)_2_), 1.71 (s, 9 H, C(6)-O-C(CH_3_)_3_), 2.27 (s, 3 H, C(2′)-SOAc), 4.04 (m, 3 H, H1-C(5′), H2-C(5′), H-C(4′)), 4.60 (triplettoid, 1 H, H-C(2′)), 4.84 (dd, *J*=7.31, *J*=4.73 Hz, 1 H, H-C(3′)), 6.11 (d, *J*=6.09 Hz, 1 H, H-C(1′)), 8.11 ppm (s, 1 H, H-C(8)); ^13^C NMR (300 MHz, CDCl_3_): *δ=*12.74–13.47 (((CH_3_)_2_*C*H)_2_Si), 17.00–17.59 (((*C*H_3_)_2_CH)_2_Si), 27.95 (N((CO)OC(*C*H_3_)_3_)_2_), 28.43 (O-C(*C*H_3_)_3_), 30.43 (SCO*C*H_3_), 50.98 (C(2′), 63.06 (C(5′)), 71.86 (C(3′)), 85.56 (C(4′)), 87.41 (C(1′)), 140.64 ppm (C(8)); ESI-MS (*m*/*z*): [*M*+H]^+^ calcd for C_38_H_65_N_5_O_10_SSi_2_ 839.41, found 840.18.

### *O*^*6*^-*tert*-Butyl-*N,N*-bis(*tert*-butyloxycarbonyl)-3′,5′-*O*-(1,1,3,3-tetraisopropylsiloxane-1,3-diyl)-2′-sulfanyl-2′-deoxyguanosine (G3)

Compound **G2** (5.04 g, 6.0 mmol) was dissolved in absolute ethanol (70 mL) and CH_2_Cl_2_ (3 mL) and was stirred for one hour at 0 °C and then treated with methyl amine (24 mL, 7 M in EtOH). After 25 min the solvents were evaporated under reduced pressure and the crude product was purified by column chromatography on SiO_2_ (0–2 % CH_3_OH in dichloromethane v/v) to yield **G3** as a red foam (4.05 g, 5.08 mmol, 85 %). TLC (3 % CH_3_OH in CH_2_Cl_2_) *R*_f_=0.79. ^1^H NMR (300 MHz, [D_6_]DMSO): *δ=*1.04 (m, 28 H, 2× (((CH_3_)_2_CH)_2_Si), 1.40 (s, 18 H, C(2)-N(Boc)_2_), 1.65 (s, 9 H, C(6)-O-C(CH_3_)_3_), 2.73 (d, *J*=8.13 Hz, 3 H, C(2′)-SH), 3.93–4.05 (m, 3 H, H1-C(5′), H2-C(5′), H-C(4′)), 4.35 (dd, 1 H, H-C(2′)), 4.62 (m, 1 H, H-C(3′)), 5.95 (d, *J*=7.20 Hz, 1 H, H-C(1′)), 8.52 ppm (s, 1 H, H-C(8)); ^13^C NMR (75 MHz, [D_6_]DMSO): *δ=*12.60–13.49 (((CH_3_)_2_*C*H)_2_Si), 17.33–17.93 (((*C*H_3_)_2_CH)_2_Si), 27.93 (N((CO)OC(*C*H_3_)_3_)_2_), 28.57 (O-C(*C*H_3_)_3_), 44.75 (C(2)), 63.62 (C(5′)), 72.84 (C(3′)), 85.67 (C(4′)), 89.96 (C(1′)), 142.99 ppm (C(8)); ESI-MS: (*m*/*z*) [*M*+H]^+^ calcd for C_36_H_64_N_5_O_9_SSi_2_ 798.40, found 798.10.

### *O*^*6*^-*tert*-Butyl-*N,N*-bis(*tert*-butyloxycarbonyl)-3′,5′-*O*-(1,1,3,3-tetraisopropylsiloxane-1,3-diyl)-2′-trifluoromethylthio-2′-deoxyguanosine (G4)

Nucleoside **G3** (562 mg, 0.704 mmol) was dissolved in dichloromethane (12 mL) and cooled to −78 °C. To this solution, 3,3-dimethyl-1-(trifluoromethyl)-1,2-benziodoxole (Togni reagent, 279 mg, 8.45 mmol) was added as solid and the mixture was stirred, overnight. Within this time, the reaction mixture was slowly allowed to warm to room temperature. The solvent was removed and the crude product was purified by column chromatography on SiO_2_ (0–1.5 % CH_3_OH in dichloromethane) yielding **G4** as a yellow foam (444 mg, 0.513 mmol, 73 %). TLC (6 % CH_3_OH in CH_2_Cl_2_ v/v) *R*_f_=0.89. ^1^H NMR (300 MHz, CDCl_3_): *δ=*1.08 (m, 28 H, 2×((CH_3_)_2_CH)_2_Si), 1.36 (s, 18 H, C(2)-N(Boc)_2_), 1.72 (s, 9 H, C(6)-OC(CH_3_)_3_), 4.03 (m, 3 H, H1-C(5′), H2-C(5′), H-C(4′)), 4.89 (m, 2 H, H-C(3′), H-C(2′)), 6.22 (d, *J*=6.39 Hz, 1 H, H-C(1′)), 7.98 ppm (s, 1 H, H-C(8)); ^13^C NMR (75 MHz, CDCl_3_): *δ=*13.47–13.54 (((CH_3_)_2_*C*H)_2_Si), 16.95–17.61 (((*C*H_3_)_2_CH)_2_Si), 27.94 (N((CO)OC(*C*H_3_)_3_)_2_), 28.38 (O-C(*C*H_3_)_3_), 49.76 (C(2′), 63.42 (C(5′)), 73.13 (C(3′)), 85.81 (C(4′)), 90.37 (C(1′)), 141.66 ppm (C(8)); ^19^F NMR (565 MHz, CDCl_3_): *δ=*−39.35 ppm; ESI-MS (*m*/*z*): [*M*+NEt_3_+H]^+^ calcd for C_43_H_78_F_3_N_6_O_9_SSi_2_ 967.50, found 967.30.

### *O*^*6*^-*tert*-Butyl-*N,N*-bis(*tert*-butyloxycarbonyl)-2′-trifluoromethylthio-2′-deoxyguanosine (G5)

Compound **G4** (908 mg, 1.05 mmol) was added to a solution of TBAF in THF (1 M; 5 mL) and was stirred at room temperature for 3 h. After that time, the solvent was removed and the crude product was purified by column chromatography on SiO_2_ (0–1.5 % CH_3_OH in dichloromethane) yielding **G5** as a yellow foam (528 mg, 0.847 mmol, 81 %). TLC (6 % CH_3_OH in CH_2_Cl_2_ v/v) *R*_f_=0.49. ^1^H NMR (300 MHz, [D_6_]DMSO): *δ=*1.34 (s, 18 H, C(2)-N(Boc)_2_), 1.65 (s, 9 H, C(6)-OC(CH_3_)_3_), 3.57, 3.69 (m, 2 H, H1-C(5′), H2-C(5′)), 4.09 (triplettoid, 1 H, H-C(4′)), 5.05 (triplettoid, 1 H, H-C(3′)), 4.73, 4.76 (dd, *J*=5.04, 4.91 Hz, 1 H, H-C(2′)), 5.19 (t, *J*=5.55 Hz, 1 H, HO-C(5′)), 6.32 (d, *J*=8.79 Hz, 1 H, H-C(1′)), 6.55 (d, *J*=5.37 Hz, 1 H, HO-C(3′)), 8.67 ppm (s, 1 H, H-C(8)); ^13^C NMR (75 MHz, [D_6_]DMSO): *δ=*27.92 (N((CO)OC(*C*H_3_)_3_)_2_), 28.37 (O-C(*C*H_3_)_3_), 50.57, 74.14 (C(2′), C(3′)), 63.20 (C(5′)), 88.91 (C(4′)), 91.75 (C(1′)), 130.22 (q, *J*=307.00 Hz, CF_3_), 143.03 ppm (C(8)); ^19^F NMR (565 MHz, CDCl_3_) *δ=*−40.00 ppm; ESI-MS (*m*/*z*): [*M*+H]^+^ calcd for C_25_H_37_F_3_N_5_O_8_S 624.23, found 624.29.

### *N*^*2*^-(*N*,*N*-Dimethylformimidamide)-2′-trifluoromethylthio-5′-*O*-(4,4′-dimethoxytriphenylmethyl)-2′-deoxyguanosine (G6)

Nucleoside **G5** (262 mg, 0.420 mmol) was dissolved in dry dichloromethane (6.7 mL), treated with trifluoroacetic acid (670 μL), and stirred at room temperature. Within few minutes, the yellow solution turned red. After 2.5 h, a second portion of trifluoroacetic acid (300 μL) was added and stirring of the solution was continued for two more hours. The reaction was stopped by addition of isopropanol (4 mL). The reaction mixture was evaporated, co-evaporated three times with isopropanol and subsequently exposed to high vacuum for 30 min. To the solid residue, methanol (10 mL) and *N*,*N*-dimethylformamide dimethyl acetal (1.5 mL, 11.3 mmol) was added. The reaction mixture was refluxed for 6 h, then evaporated to dryness, and extensively dried under high vacuum. Then, 4,4′-dimethoxytrityl chloride (156 mg, 0.460 mmol), and 4-(dimethylamino)-pyridine (approximately 5 mg) were dissolved in pyridine (2 mL) and added to the solid from above, yielding a suspension that was stirred for 18 h. Methanol (1 mL) was added, the solvents evaporated and the residue co-evaporated with methanol. The crude product was purified by column chromatography on SiO_2_ (0.5–3 % CH_3_OH in dichloromethane containing 0.5 % triethylamine) yielding **G6** as yellow foam (186 mg, 0.202 mmol, 48 %). TLC (6 % CH_3_OH in CH_2_Cl_2_ v/v) *R*_f_=0.16. ^1^H NMR (300 MHz, [D_6_]DMSO): *δ=*2.99 (s, 6 H, N(CH_3_)_2_), 3.42 (s, 2 H, H1-C(5′), H2-C(5′)), 3.74 (s, 6 H, 2×ar-O-CH_3_), 4.34 (triplettoid, 1 H, H-C(4′)), 4.47, 4.50 (dd, *J*=5.13 Hz, *J*=4.19 Hz, 1 H, H-C(2′)), 4.78 (triplettoid, 1 H, H-C(3′)), 6.78 (d, *J*=8.37 Hz, 1 H, H-C(1′)), 6.76–7.40 (m, 13 H, H-C(ar)), 7.71 (s br, 1 H, H-N(1)), 8.13 (s, 1 H, H-C(8)), 8.51 ppm (s, 1 H, H-C=N-C(6)); ^13^C NMR (75 MHz, [D_6_]DMSO): *δ=*35.23, 41.27 (N(CH_3_)_2_), 52.35 (C(2′), 63.93 (C(5′)), 72.92 (C(3′)), 85.84 (C(4′)), 89.95 (C(1′)), 113.42, 127.15–130.22(C(ar)), 149.13 ppm (C(8)); ^19^F NMR (565 MHz, [D_6_]DMSO): *δ=*−73.51 ppm; ESI-MS (*m*/*z*): [*M*+NEt_3_+H]^+^ calcd for C_41_H_50_F_3_N_7_O_6_S 825.35, found 826.13.

### *N*^*2*^-[(Dimethylamino)methylene]-2′-trifluoromethylthio-5′-*O*-(4,4′-dimethoxytriphenylmethyl)-2′-deoxyguanosine 3′-*O*-((2-cyanoethyl) *N*,*N*-diisopropylphosphoramidite) (G7)

Nucleoside **G6** (167 mg, 0.230 mmol) was dissolved in absolute dichloromethane (2.0 mL) and ethyldimethyl amine (175 μL, 1.612 mmol) and stirred for 15 min before 2-cyanoethyl-*N,N*-diisopropylchlorophosphoramidite (111 mg, 0.469 mmol) was added dropwise. The reaction was monitored by thin layer chromatography which showed almost complete reaction after 1.5 h, more 2-cyanoethyl-*N,N*-diisopropylchlorophosphoramidite (40 mg, 0.169 mmol) was added slowly. After a total reaction time of 2.5 h, the reaction was quenched by the addition of CH_3_OH (50 μL) and stirring was continued for seven more minutes before the solvents were evaporated and dried under high vacuum to give a slightly yellow foam. The crude product was subjected to column chromatographic purification on SiO_2_ (ethylacetate/hexane 4:6 to 100 % ethylacetate, then 1 % CH_3_OH in ethylacetate; all eluents contained 0.5 % triethylamine) yielding **G7** as white foam (154 mg, 0.166 mmol, 72 %). TLC (6 % CH_3_OH in CH_2_Cl_2_+1 % triethylamine v/v) *R*_f_=0. 35. ^1^H NMR (300 MHz, CDCl_3_): *δ=*1.08–1.26 (m, 12 H, PN(CH(C*H_3_*)_2_)_2_), 2.35, 2.63 (2×m, 4 H, 2×OCH_2_C*H_2_*CN), 3.05 (m, 12 H, 2×N(CH_3_)_2_), 3.62 (m, 2 H, OC*H*_2_CH_2_CN), 3.92 (m, 2 H, OC*H_2_*CH_2_CN), 3.78 (s, 6 H, 2×ar-O-CH_3_), 4.32, 4.41 (2×s, 2 H, 2×H-C(4′)), 4.51–4.78 (m 4 H, 2×H-C(3′), 2×H-C(2′)), 6.17, 6.23 (2×d, *J*=9.15, 8.88 Hz); 6.80–7–39 (m, 13 H, H-C(ar)), 7.73, 7.76 (2×s, 2 H, 2×H-C(8)), 8.46, 8.51 (2×s, 2 H, 2×H-C=N-C(6)), 9.35 ppm (s br, H-N(1)); ^13^C NMR (75 MHz, CDCl_3_): *δ=*20.24–20.63 (*C*H_2_CN), 24.72 (N(CH_3_)_2_, PN(CH(C*H_3_*)_2_)_2_), 43.46–43.67 (O-CH_2_*C*H_2_-CN), 55.40 (O-CH_3_), 57.98–63.58 (C(5′), O-*C*H_2_CH_2_-CN), 75.10; 74.87 (C(3′)), 75.91, 76.11 (C(2′)), 84.71, 84.98 (2× C(4′)), 87.07, 87.21 (2×C(1′)); 113.50, 127.30, 128.22, 130.18 ppm (C(ar)), 158.24 H-C=N); ^19^F NMR (565 MHz, CDCl_3_): *δ=*−39.64, −39.82 ppm; ^31^P NMR (121 MHz, CDCl_3_): *δ=*151.27, 152.47 ppm; ESI-MS (*m*/*z*): [*M*+H]^+^ calcd for C_44_H_53_F_3_N_8_O_7_PS 925.34, found 925.25.

### Solid-phase RNA synthesis

All oligonucleotides were synthesized on a 1.0 μmol scale using an Applied Biosystems ABI392, following standard synthesis protocols. Detritylation (2 min): 4 % (v/v) dichloroacetic acid in 1,2-dichloroethane. Coupling (3 min): 120 μL phosphoramidite (0.1 M) in acetonitrile plus 360 μL 5-(benzylthio)-1*H*-tetrazole in acetonitrile (0.30 M) as activator. Capping (2×0.5 min): 1:1 (v/v) Cap A/Cap B, Cap A: 0.2 M phenoxyacetic anhydride in dry THF, Cap B: 1-methyl imidazole (0.2 M) and 2,4,6-trimethylpyridine (0.2 M) in dry THF. Oxidation (1 min): 20 mM iodine in 7:2:1 (v/v/v) THF/pyridine/water. Phosphoramidite and activator solutions were dried over activated molecular sieves (3 Å), overnight. All sequences were synthesized trityl-off.

### Deprotection of 2′-SCF_3_-containing RNA

The solid support was transferred into a screw-capped Eppendorf tube and 1.5 mL of a 1:1 mixture of solution of methylamine in ethanol (33 %) ammonia and aqueous methylamine (40 %) was added and the reaction proceeded at room temperature for 4 to 6 h with occasional shaking. IMPORTANT: For deprotection of 2′-SCF_3_ adenosine containing RNA, the deprotection solution additionally contained 150 mM of threo-1,4-dimercapto-2,3-butandiol (DTT).[26,33] The suspension was filtered and all volatiles evaporated. The residue was dissolved in 1.0 mL of 1 M tetrabutylammonium fluoride trihydrate in THF and kept at 37 °C for 12 h. The reaction was quenched by addition of 1.0 mL of triethylammonium bicarbonate buffer (1 M, pH 7.4) and the organic solvent was evaporated. The solution was subjected to size-exclusion chromatography on an Amersham HiPrep 26/10 desalting column (2.6×10 cm; Sephadex G25). The crude RNA was eluted with H_2_O, dried, and redissolved in 1 mL H_2_O.

### Analysis and purification of 2′-SCF_3_-containing RNA

Analysis of crude products was performed by anion-exchange HPLC on a Dionex DNAPac-100 column (4×250 mm) at 80 °C. Flow rate: 1 mL min^−1^; eluant A: 25 mM Tris-HCl (pH 8.0), 6 M urea; eluant B: 25 mM Tris-HCl (pH 8.0), 6 M urea, 500 mM NaClO_4_; gradient: 0–40 % B in A within 25 min; UV detection at 260 nm. Crude products were purified on a semipreparative Dionex DNAPac-100 column (9×250 mm) at 80 °C. Flow rate: 2 mL min^−1^; gradient: 3–17 % B in A within 15 min for oligonucleotides <10 nt; 24–35 % B in A within 18 min for oligonucleotides >10 nt; UV detection at 260 nm. Fractions containing the oligonucleotide were diluted with an equal volume of triethylammonium bicarbonate buffer (100 mM, pH 7.4) and loaded on an equilibrated C18 SepPak Plus cartridge (Waters), washed with water and eluted with water/acetonitrile (1:1, v/v). Purified fractions were evaporated and redissolved in 1.0 mL water. The RNA yield was determined as units of optical density at 260 nm by UV spectroscopy (Implen NanoPhotometer) at room temperature. The product quality and purity was verified by anion-exchange chromatography on an analytical column (vide supra).

### RNA interference and Northern analysis

Lyophilized synthetic siRNA duplexes were dissolved, annealed, and delivered into chicken DF-1 cells by electroporation as described.[45] Total RNA isolation, and analysis of gene silencing by Northern hybridization using specific ^32^P-radiolabelled DNA probes for detection of *BASP1* and *GAPDH* mRNAs were done as described previously.[45,46]
