# Supplementary material for: Expanding the Scope of 2′-SCF3 Modified RNA
Source: Chemistry. 2015 Jun 12;21(29):10400–7. doi: 10.1002/chem.201500415 (PMC4515092; doi:10.1002/chem.201500415)
Supplement: Supplementary file 1 [file chem0021-10400-sd1.pdf]

CHEMISTRY
A European Journal

Supporting Information

# Expanding the Scope of 2′-$SCF_3$ Modified RNA

Lukas Jud,[a] Marija Košutić,[a] Veronika Schwarz,[a] Markus Hartl,[b] Christoph Kreutz,[a] Klaus Bister,[b] and Ronald Micura*[a]

chem_201500415_sm_miscellaneous_information.pdf

*Contents*


**1. Synthesis of 2'-deoxy-2'-trifluoromethylthioadenosine building block A9 (Procedures and NMR spectra) 2**

– General 2

– *N*6-[(Dibutylamino)methylene]-3',5'-*O*-(1,1,3,3-tetra*iso*propylsiloxane-1,3-diyl) (β-D-arabinofuranosyl)adenine (**A2**) 2

– *N*6-[(Dibutylamino)methylene]-3',5'-*O*-(1,1,3,3-tetraisopropylsiloxane-1,3-diyl)-2'-*O*-(trifluoromethanesulfonyl) (β-D-arabinofuranosyl)adenine (**A3**) 2

– *N*6-[(Dibutylamino)methylene]-3',5'-*O*-(1,1,3,3-tetraisopropylsiloxane-1,3-diyl)-2'- acetylthio-2'-deoxyadenosine (**A4**) 2

– *N*6-[(Dibutylamino)methylene]-3',5'-*O*-(1,1,3,3-tetraisopropylsiloxane-1,3-diyl)-2'-sulfanyl-2'-deoxyadenosine (**A5**) 3

– *N*6-[(Dibutylamino)methylene]-3',5'-*O*-(1,1,3,3-tetraisopropylsiloxane-1,3-diyl) -2'-trifluoromethylthio-2'-deoxyadenosine (**A6**) 4

– *N*6-[(Dibutylamino)methylene]-2'-trifluoromethylthio-2'-deoxy-adenosine (**A7**) 6

– *N*6-[(Dibutylamino)methylene]-2'-trifluoromethylthio-5'-*O*-(4,4'-dimethoxytrityl)-2'-deoxyadenosine (**A8**) 7

– *N*6-[(Dibutylamino)methylene]-2'-trifluoromethylthio-5'-*O*-(4,4'-dimethoxytrityl)-2'-deoxyadenosine 3'-O-(2-cyanoethyl *N*,*N*-diisopropylphosphoramidite) (**A9**) 9

**2. NMR spectra of 2'-SCF$_3$ guanosine building block G7 and the precursors G1 to G6 10**

– *O*6-*tert*-Butyl-*N,N*-bis(*tert*-butyloxycarbonyl)-3',5'-*O*-(1,1,3,3-tetraisopropylsiloxane-1,3-diyl)-arabinoguanosine (**G1**) 10

– *O*6-*tert*-Butyl-*N,N*-bis(*tert*-butyloxycarbonyl)-3',5'-*O*-(1,1,3,3-tetraisopropylsiloxane-1,3-diyl)-2'-acetylthio-2'-deoxyguanosine (**G2**) 11

– *O*6-*tert*-Butyl-*N,N*-bis(*tert*-butyloxycarbonyl)-3',5'-*O*-(1,1,3,3-tetraisopropylsiloxane-1,3-diyl)-2'-sulfanyl-2'-deoxyguanosine (**G3**) 12

– *O*6-*tert*-Butyl-*N,N*-bis(*tert*-butyloxycarbonyl)-3',5'-*O*-(1,1,3,3-tetraisopropylsiloxane-1,3-diyl)-2'-trifluoromethylthio-2'-deoxyguanosine (**G4**) 13

– *O*6-*tert*-Butyl-*N,N*-bis(*tert*-butyloxycarbonyl)-2'-trifluoromethylthio-2'-deoxyguanosine (**G5**) 14

– *N*2-[(Dimethylamino)methylene]-2'-trifluoromethylthio-5'-*O*-(4,4'- dimethoxytrityl)-2'-deoxyguanosine (**G6**) 15

– *N*2-[(Dimethylamino)methylene]-2'-trifluoromethylthio-5'-*O*-(4,4'-dimethoxytrityl)-2'-deoxyguanosine 3'-*O*-((2-cyanoethyl) *N*,*N*-diisopropylphosphoramidite) (**G7**) 16

**3. Mass spectrometry of 2'-SCF$_3$ modified RNA 17**

**4. $^{19}$F and $^{1}$H NMR experiments of 2'-SCF$_3$ modified RNA 17**

**5. UV-melting profile experiments and analysis 17**

**Table S1**. Selection of 2'-SCF$_3$ modified RNAs synthesized in the course of this study 18

**Table S2**. Selection of 2'-SCF$_3$ modified siRNAs investigated in this study 19

**Supporting Figure S1**. Thermal stabilities of a 2'-SCF$_3$ uridine modified DNA hairpin. 21

**Supporting Figure S2**. Duplex formation of 2'-SCF$_3$ adenosine modified RNA. 20

**Supporting Figure S3**. Thermodynamic analysis of siRNA duplexes. 22

## 1. Synthesis of 2′-deoxy-2′-trifluoromethylthioadenosine building block A9.

**General.** $^{1}H$, $^{13}C$, $^{31}P$ and $^{19}F$ NMR spectra were recorded on a Bruker DRX 300 MHz or Avance II+ 600 MHz instrument. Chemical shifts are referenced to the deuterated solvents ($CDCl_3$ (7.26 ppm - $^{1}H$; 77.1 ppm - $^{13}C$); $d_6$-DMSO (2.5 ppm - $^{1}H$; 39.5 ppm - $^{13}C$); $^{31}P$ shifts are relative to external 85 % phosphoric acid, $^{19}F$ shifts are relative to external $CCl_3F$). Assignment of $^{1}H$, $^{13}C$ spectra were carried out using COSY and HSQC experiments. MS experiments were performed on a Finnigan LCQ Advantage MAX ion trap instrument. Unless specified, all reactions were carried out at room temperature and under argon atmosphere. POLYGRAM® SIL G/$UV_{254}$ pre-coated polyester sheets (0.2 mm silica gel with fluorescent indicator) were used for thin layer chromatography. The nucleosides were visualized at 254 nm. Column chromatography was executed on silica gel purchased from Fluka (pore size 60 Å, 70 – 230 mesh, 63 – 200 µm). 1 % Triethylamine was added packing silica gel columns. Chemical reagents and solvents were purchased from commercial suppliers and used without further purification. Organic solvents for reactions were dried overnight over freshly activated molecular sieves (4 Å).

***N*$^6$-[(Dibutylamino)methylene]-3′,5′-*O*-(1,1,3,3-tetraisopropylsiloxane-1,3-diyl) (β-D-arabino-furanosyl)adenine (A2).** β-D-Arabinofuranosyladenine (2.35 g, 8.79 mmol) was coevaporated three times with pyridine. DMF/pyridine (1/1, 50 mL) was added resulting a suspension to which 1,3-dichloro-1,1,3,3-tetraisopropyldisiloxane (3.48 mL, 10.87 mmol) was added dropwise. The suspension turned into a transparent solution during stirring for 24 h. The solvents were removed under reduced pressure, the residue dissolved in dichloromethane and sequentially washed with aqueous $NaHCO_3$ (5%) and brine, dried over $Na_2SO_4$, evaporated, and dried under vacuum yielding **A1** as colorless oil which was used for the next step without further purification. TLC (6% $CH_3OH$ in dichloromethane) $R_f$ = 0.45. Nucleoside **A1** (4.85 g, 9.51 mmol) was dissolved in DMF (53 mL), *N*,*N*-dibutylformamid-dimethylacetal (4.57 g, 22.5 mmol) was added and the solution was stirred for 26 h. After that time, the DMF was removed under reduced pressure yielding a yellow oil, which was purified by column chromatographic purification on $SiO_2$ (0.5–3.5% $CH_3OH$ in dichloromethane) yielding **A2** (5.20 g, 96% over two steps) as a colorless foam. TLC (6% $CH_3OH$ in dichloromethane) $R_f$ = 0.24. $^{1}H$-NMR (300 MHz, $CDCl_3$): δ 0.95 (m, 6H, $N(CH_2CH_2CH_2CH_3)_2$); 1.04 - 1.09 (m, 28H, 2x $((CH_3)_2CH)_2Si$); 1.34 (m, 4H, $N(CH_2CH_2CH_2CH_3)_2$); 1.63 (m, 4H, $N(CH_2CH_2CH_2CH_3)_2$); 3.37 (t, *J* = 6.95 Hz, 2H, $N(CH_2CH_2CH_2CH_3)_2$); 3.57 (m, 1H, H-C(3′)); 3.94 (m, 2H, $N(CH_2CH_2CH_2CH_3)_2$; 3.99 (m, 3H, H1-C(5′), H2-C(5′), H-C(4′)); 4.64 (m, 1H, H-C(2′)); 5.46 (s, br, HO-C(2′)); 6.16 ((d, *J* = 4.89 Hz, H-C(1′)); 8.02 (s, 1H, H-C(8)); 8.30 (s, 1H, H-C(2)); 8.79 (s, 1H, H-C=N-C(6)) ppm. ESI-MS (m/z): $[M+H]^+$ calcd for $C_{31}H_{57}N_6O_5Si_2$ 649.38, found 649.42.

***N*$^6$-[(Dibutylamino)methylene]-3′,5′-*O*-(1,1,3,3-tetraisopropylsiloxane-1,3-diyl)-2′-*O*-(trifluoro-methanesulfonyl) (β-D-arabinofuranosyl)adenine (A3).** Compound **A2** (5.43 g, 8.37 mmol) was dissolved in dry dichloromethane (100 mL) and the solution was cooled to 0°C. Then, 4-(*N*,*N*-dimethylamino)pyridine (3.08 g, 25.2 mmol) followed by trifluoromethanesulfonyl chloride (1.33 mL; 12.5 mmol) were added, and the solution was stirred for 30 min. After that time, the yellow solution was washed with 5% aqueous $NaHCO_3$ solution, dried over $Na_2SO_4$, evaporated, and dried under vacuum. The resulting compound **A3** was used for the next step without further purification. TLC (6% $CH_3OH$ in dichloromethane) $R_f$ = 0.53.

***N*$^6$-[(Dibutylamino)methylene]-3′,5′-*O*-(1,1,3,3-tetraisopropylsiloxane-1,3-diyl)-2′-acetylthio-2′-deoxyadenosine (A4).** Nucleoside **A3** (6.53 g, 8.36 mmol) was dissolved in toluene (190 mL). Potassium thioacetate (1.43 g, 12.6 mmol) and 18-crown-6 (3.32 g, 12.5 mmol) were added, and the sudpension stirred at 45°C for 17 h, yielding a yellow solution. The solvents were removed under reduced pressure and the crude product purified by column chromatography on $SiO_2$ (3.5/6.5–8/2 hexan/ethylacetate v/v) yielding **A4** as yellow oil (5.49 g, 7.764 mmol, 93% over two steps). TLC (6% $CH_3OH$ in dichloromethane) $R_f$ = 0.51. $^{1}H$-NMR (300 MHz, $CDCl_3$): *δ* 0.93 (t, 6H, $N(CH_2CH_2CH_2CH_3)_2$); 1.05–1.11 (m, 28H, 2x $((CH_3)_2CH)_2Si$); 1.36 (m, 4H, $N(CH_2CH_2CH_2CH_3)_2$); 1.62 (m, 4H,

N($CH_2CH_2CH_2CH_3$)$_2$); 2.29 (s, 3H, $SCOCH_3$); 3.37 (t, 4H, N($CH_2CH_2CH_2CH_3$)$_2$); 3.69 (t, 4H, N($CH_2CH_2CH_2CH_3$)$_2$); 4.04 (m, 3H, H1-C(5'),H2-C(5'), H-C(4')); 4.70 (triplettoid, 1H, H-C(2')); 5.22 (m, 1H, H-C(3')); 6.07 (dd, 1H, H-C(1')); 8.00 (s, 1H, H-C(8)); 8.51 (s,1H, H-C(2)); 8.98 (s, 1H, C(6)N=C-H) ppm. $^{13}$C- NMR (75 MHz, $CDCl_3$): $\delta$ 13.65, 13.82 (N($CH_2CH_2CH_2CH_3$)$_2$); 17.34 (2x (($CH_3$)$_2$CH)$_2$Si); 19.67, 20.20 (N($CH_2CH_2CH_2CH_3$)$_2$); 29.46 (N($CH_2CH_2CH_2CH_3$)$_2$), 45.26, 51.90 (N($CH_2CH_2CH_2CH_3$)$_2$); 50.62 (C(2')); 62.62 (C(5')); 71.88 (C(3')); 84.71 (C(4')); 90.50 (C(1')); 140.84 (C(8)); 152,87 (C(2)) ppm. ESI-MS (m/z): $[M+H]^+$ calcd $C_{33}H_{58}N_6O_5SSi_2$ 707.37, found 707.42.

$^1$H-NMR (300 MHz, $CDCl_3$) of **A4**

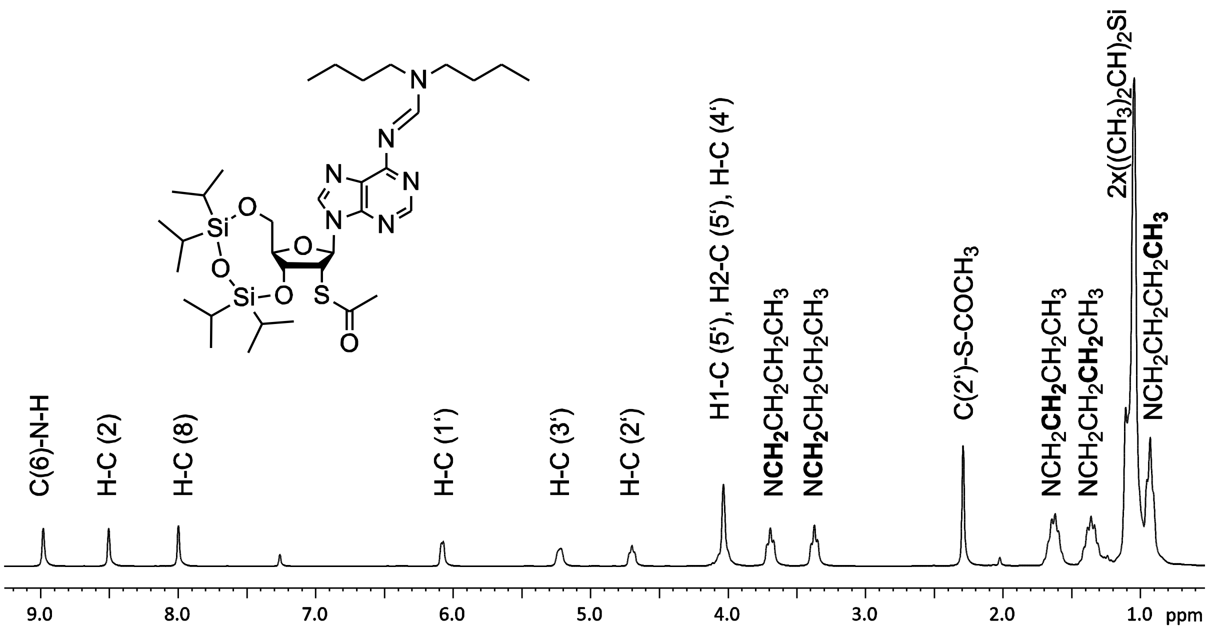


$^{13}$C-NMR (75 MHz, $CDCl_3$) compound **A4**

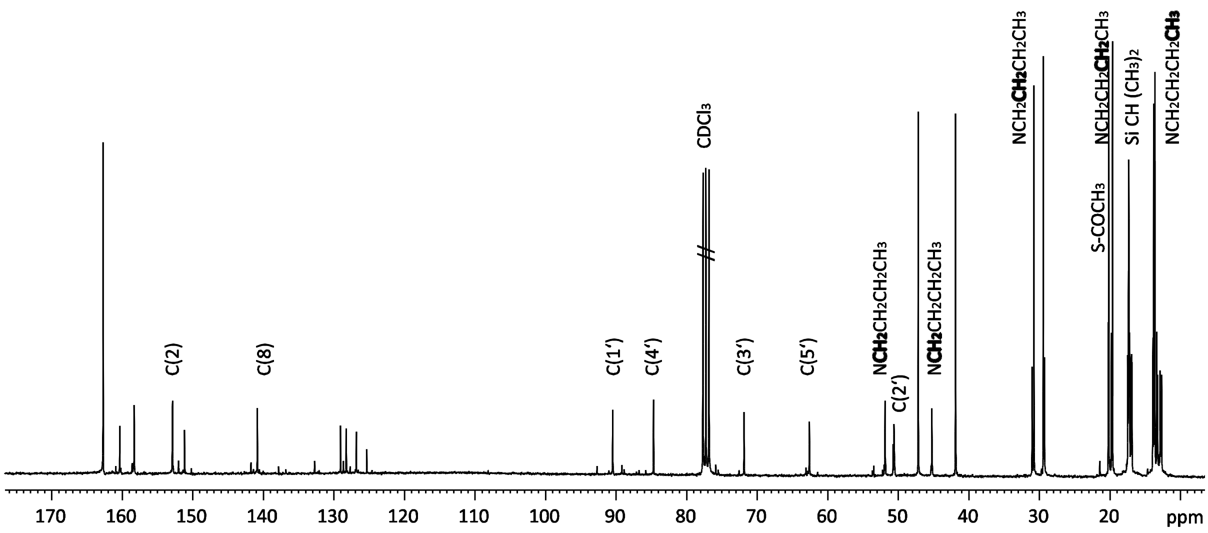


***N*$^6$-[(Dibutylamino)methylene]-3',5'-*O*-(1,1,3,3-tetraisopropylsiloxane-1,3-diyl)-2'-sulfanyl-2'-deoxyadenosine (A5).** Nucleoside **A4** (0.20 g, 0.28 mmol) was dissolved in ethanol/pyridine (1/1, 20 mL) and the solution was cooled to 0°C. A solution of 1 M NaOH in Ethanol/$H_2O$ (1/1, 2.86 mL) was added. After 10 min, DOWEX® was added until the pH value of the solution became neutral. DOWEX® was removed by filtration, the solvents were evaporated, and the crude product purified by column chromatography on $SiO_2$ (0.5–2.5% $CH_3OH$ in dichloromethane) yielding **A5** as colorless oil (140 mg, 0.211 mmol, 75%). TLC (6% $CH_3OH$ in dichloromethane) $R_f$ = 0.42. $^1$H NMR (300 MHz, $d_6$-DMSO): $\delta$

0.93 (t, 6H, N($CH_2CH_2CH_2CH_3$)$_2$); 1.08 (m, 28H, 2x (($CH_3$)$_2$CH)$_2$Si); 1.30–1.32 (m, 4H, N($CH_2CH_2CH_2CH_3$)$_2$); 1.60 (m, 4H, N($CH_2CH_2CH_2CH_3$)$_2$); 2.72 (d, 1H, C(2')SH); 3.45 (m, 4H, N($CH_2CH_2CH_2CH_3$)$_2$); 3.57 (m, 4H, N($CH_2CH_2CH_2CH_3$)$_2$); 3.98(m, 2H, H1-C(5'),H2-C(5')); 4.03 (m, 1H, H-C(4')); 4.41–4.43 (dd, 1H, H-C(2')); 4.88 (triplettoid, 1H, H-C(3')); 6.03 (dd, 1H, H-C(1')); 8.40 (s, 2H, H-C(8), C(6)N-H); 8.92(s, 1H, H-C(2)) ppm. $^{13}$C NMR (75 MHz, $d_6$-DMSO): $\delta$ 12.67 (N($CH_2CH_2CH_2CH_3$)$_2$); 14.06 (2x (($CH_3$)$_2$CH)$_2$Si); 17.77 (N($CH_2CH_2CH_2CH_3$)$_2$); 20.15 (N($CH_2CH_2CH_2CH_3$)$_2$), 40.26, 46.50 (N($CH_2CH_2CH_2CH_3$)$_2$); 45.11 (C(2')); 63.68 (C(5')); 72.36 (C(3')); 84.91 (C(4')); 90.30 (C(1')); 153.30 (C(8)); 160.73 (C(2)) ppm. ESI-MS (m/z): $[M+H]^+$ calcd for $C_{31}H_{57}N_6O_4SSi_2$ 665.36, found 665.44.

$^1$H NMR (300 MHz, $d_6$-DMSO) of **A5**

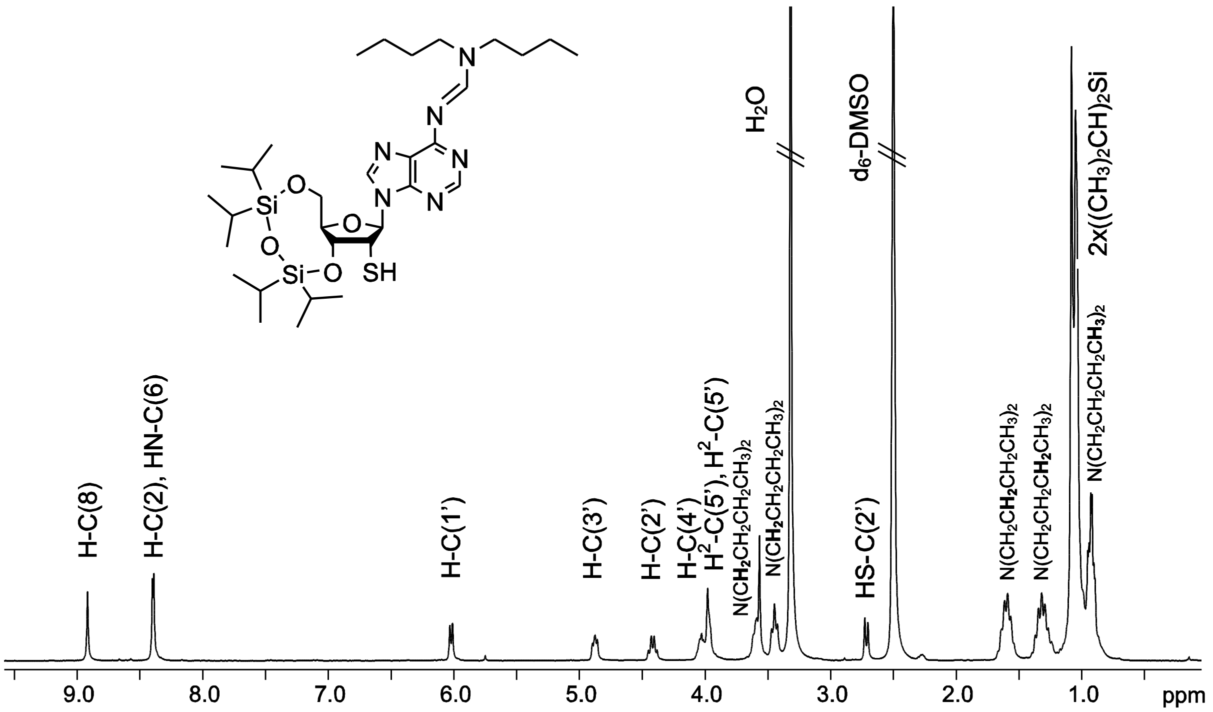


$^{13}$C NMR (75 MHz, $d_6$-DMSO) of **A5**

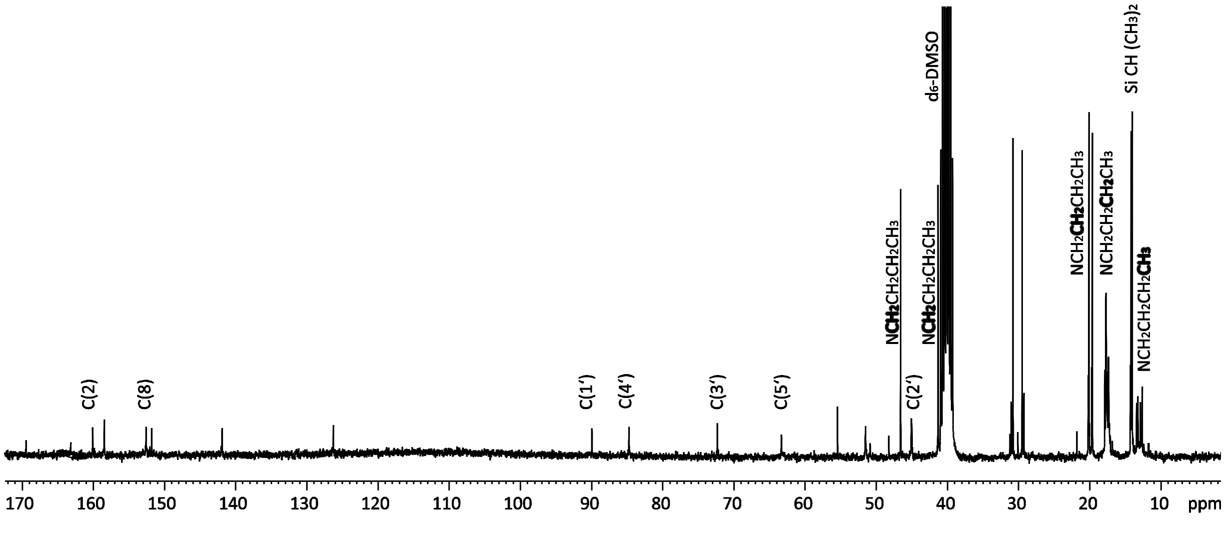


***N*$^6$-[(Dibutylamino)methylene]-3',5'-*O*-(1,1,3,3-tetraisopropylsiloxane-1,3-diyl)-2'-trifluoro-methylthio-2'-deoxyadenosine (A6).** Compound **A5** (1.50 g, 2.26 mmol) was dissolved in dichloromethane (45 mL) and the solution was cooled to –78°C. 3,3-Dimethyl-1-(trifluoromethyl)-1,2-benziodoxole (Togni's reagent; 0.89 g, 2.70 mmol) was added as solid. The stirred solution was

allowed to warm to room temperature and stirring continued for 16 h. The solvents were evaporated and the crude product purified by column chromatography on $SiO_2$ (0.5–2.0% $CH_3OH$ in dichloromethane) yielding **A6** (1.48 g, 85%) as slightly yellow oil. TLC (6% $CH_3OH$ in dichloromethane v/v) $R_f$ = 0.67. $^1$H NMR (300 MHz, $CDCl_3$): $\delta$ 0.91–0.92 (m, 6H, $N(CH_2CH_2CH_2CH_3)_2$); 1.05–1.10 (m, 28H, 2x $((CH_3)_2CH)_2Si$); 1.31 (m, 2H, $N(CH_2CH_2CH_2CH_3)_2$); 1.50 (m, 2H, $N(CH_2CH_2CH_2CH_3)_2$); 3.17 (t, *J* = 7.11 Hz, 2H, $N(CH_2CH_2CH_2CH_3)_2$); 3.27 (t, *J* = 7.44, 2H, $N(CH_2CH_2CH_2CH_3)_2$); 3.37 (t, *J* = 7.28 Hz, 2H, $N(CH_2CH_2CH_2CH_3)_2$); 3.69 (t, *J* = 7.47 Hz, 2H, $N(CH_2CH_2CH_2CH_3)_2$); 4.04 (s, 3H, H1-C(5'), H2-C(5'), H-C(4')); 4.88 (dd, *J* = 7.79, 4.94 Hz, 1H, H-C(2')); 5.28 (m, 1H, H-C(3')); 6.20 (d, *J* = 4.89 Hz, 1H, H-C(1')); 7.90 (s, 1H, H-C(8)); 8.48 (s, 1H, H-C(2)); 8.95 (s, 1H, H-C=N-C(6)) ppm. $^{13}$C NMR (75 MHz, $CDCl_3$): δ 12.76–17.57, (2x $((CH_3)_2CH)_2Si$); 13.91, 20.29 ($N(CH_2CH_2CH_2CH_3)_2$); 29.53, 30.88 ($N(CH_2CH_2CH_2CH_3)_2$); 41.99, 47.29 ($N(CH_2CH_2CH_2CH_3)_2$); 45.35, 51.99 ($N(CH_2CH_2CH_2CH_3)_2$); 50.69 (C(2')); 62.66 (C(5')); 71.92 (C(3')); 84.78 (C(4')); 90.60 (C(1')); 126.90 (C(5)); 140.38 (C(8)); 152.97 (C(2)) ppm. $^{19}$F NMR (565 MHz, $CDCl_3$): δ –39.30 ppm. ESI-MS (m/z): $[M+H]^+$ calcd for $C_{32}H_{56}F_3N_6O_4SSi_2$ 733.35, found 733.37.

$^1$H NMR (300 MHz, $CDCl_3$) compound **A6**

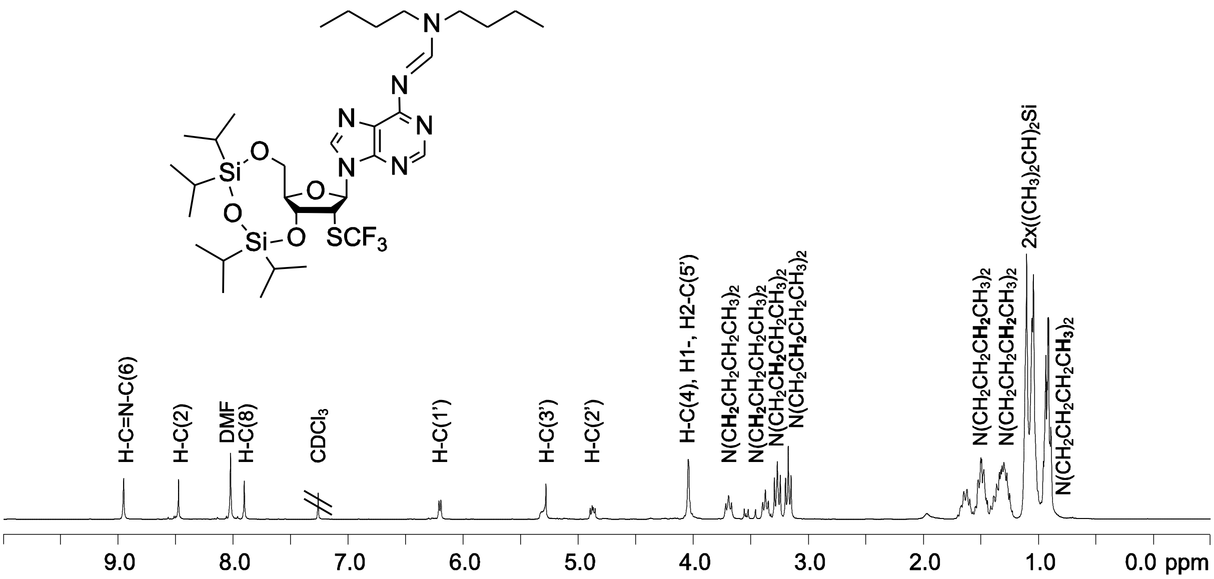


$^{13}$C NMR (75 MHz, $CDCl_3$) compound **A6**

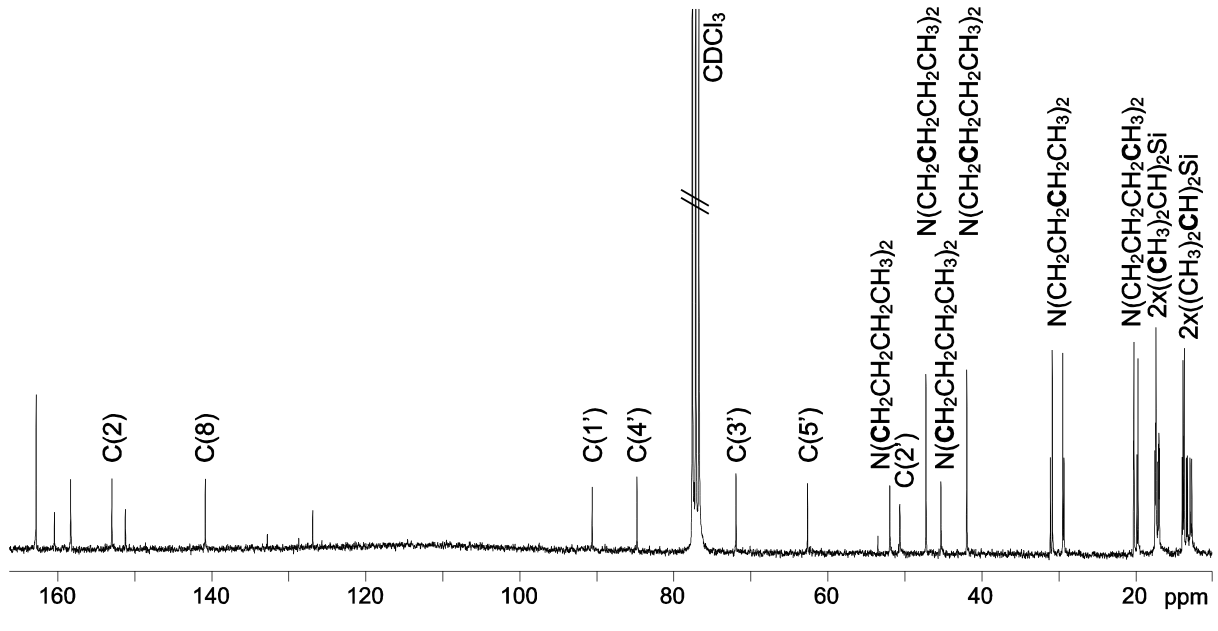

***N*[6]-[(Dibutylamino)methylene]-2′-trifluoromethylthio-2′-deoxy-adenosine (A7).** Compound **A6** (2.80 g, 3.82 mmol) was dissolved in a solution of tetrabutylammoniumfluoride trihydrate (1 M in THF, 8 mL) and acetic acid (1 M in water, 8 mL) and stirred for one hour. Then, the solvents were removed under reduced pressure. The remaining residue was coevaporated with dichloromethane twice to give a yellow oil. The crude product was purified by column chromatography on $SiO_2$ (1–4% $CH_3OH$ in dichloromethane v/v) yielding **A7** as white foam (1.26 g, 72%). TLC (10% $CH_3OH$ in dichloromethane v/v) $R_f$ = 0.44. $^1H$ NMR (300 MHz, $d_6$-DMSO): $\delta$ 0.91 (m, 6H, $N(CH_2CH_2CH_2C\mathit{H}_3)_2$); 1.31 (m, 4H, $N(CH_2CH_2C\mathit{H}_2CH_3)_2$); 1.58 (m, 4H, $N(CH_2C\mathit{H}_2CH_2CH_3)_2$); 3.35 (t, *J* = 6.95 Hz, 2H, $N(C\mathit{H}_2CH_2CH_2CH_3)_2$); 3.59 (t, *J* = 7.37 Hz, 2H, $N(C\mathit{H}_2CH_2CH_2CH_3)_2$, H1-C(5′)); 3.73 (m, 1H, H2-C(5′)); 4.12 (m, 1H, H-C(4′)); 4.50 (m, 1H, H-C(3′)); 4.80 (dd, *J* = 8.66, 5.03 Hz, 1H, H-C(2′)); 5.49 (m, 1H, HO-C(5′)); 6.35 (d, *J* = 8.86 Hz, H-C(1′)); 6.54 (d, *J* = 5.10 Hz, 1H, HO-C(3′)); 8.44 (s, 1H, H-C(8)); 8.54 (s, 1H, H-C(2)); 8.94 (s, 1H, H-C=N-C(6)) ppm. $^{13}C$ NMR (75 MHz, $d_6$-DMSO): $\delta$ 14.11, 14.30 (2x $N(CH_2CH_2CH_2\mathit{C}H_3)_2$); 19.73, 20.24 (2x $N(CH_2CH_2\mathit{C}H_2CH_3)_2$); 29.27, 31.05 (2x $N(CH_2\mathit{C}H_2CH_2CH_3)_2$); 45.12, 51.59 (2x $N(\mathit{C}H_2CH_2CH_2CH_3)_2$); 50.46 (C(2′)); 62.23 (C(5′)); 73.34 (C(3′)); 88.10 (C(4′)); 89.21 (C(1′)); 131.08 (q, *J* = 306.4 Hz, $CF_3$); 142.32 (C(2)); 152.49 (C(8)) ppm. $^{19}F$ NMR (565 MHz, $CDCl_3$) $\delta$ –40.09 ppm. ESI-MS (m/z): $[M+H]^+$ calcd for $C_{20}H_{30}F_3N_6O_3S$ 491.20, found 491.20.

$^1H$ NMR (300 MHz, $d_6$-DMSO) compound **A7**

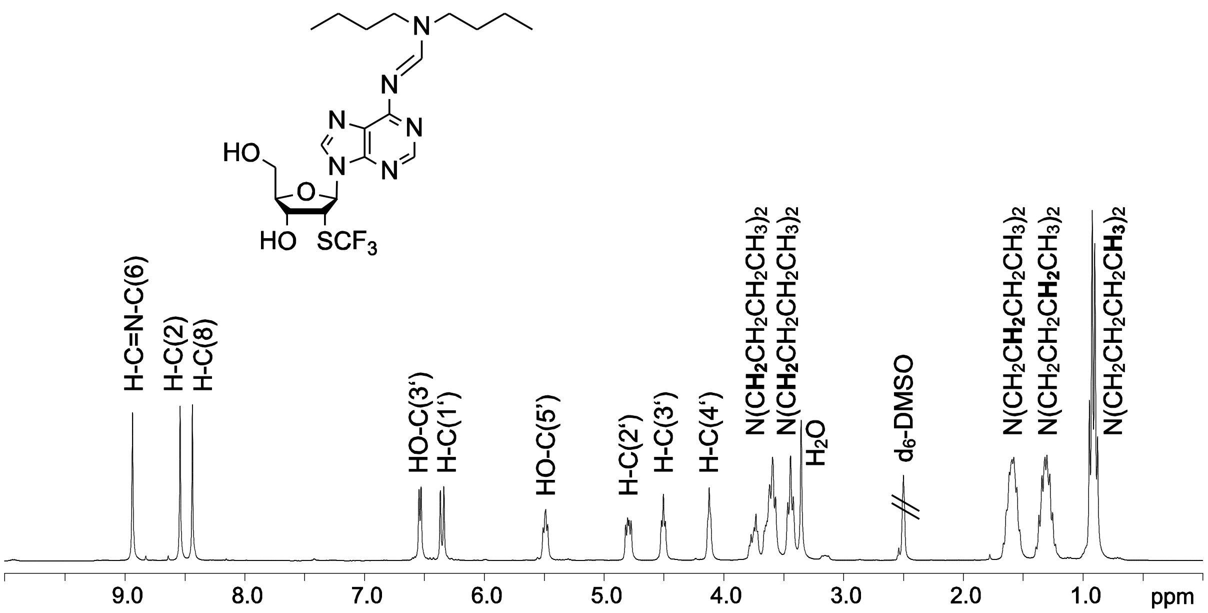


$^{13}C$ NMR (75 MHz, $d_6$-DMSO) compound **A7**

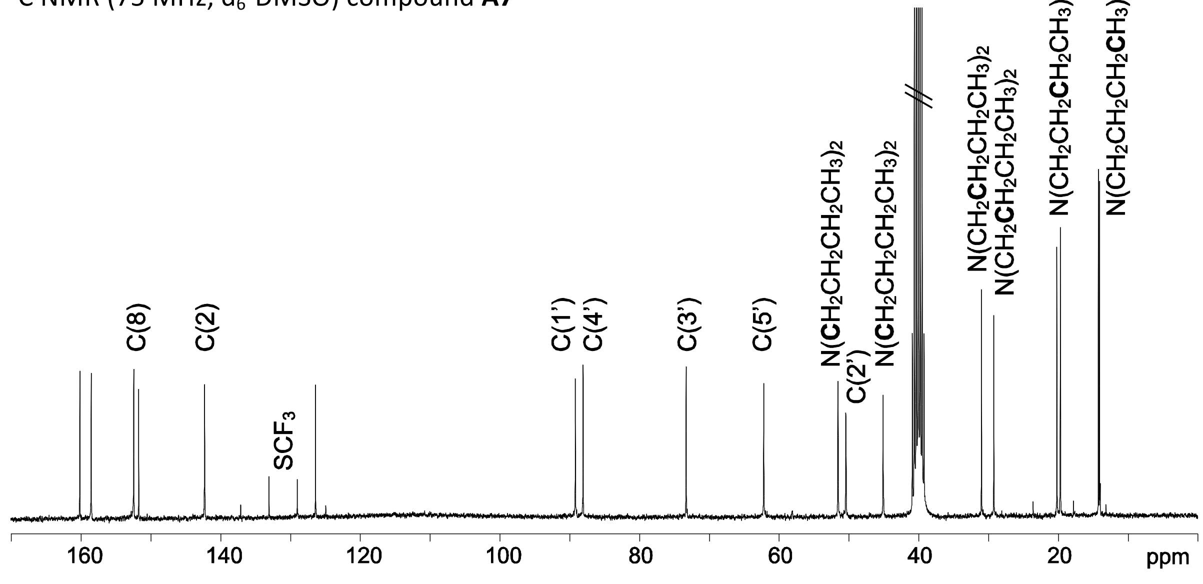

**$N^6$-[(Dibutylamino)methylene]-2'-trifluoromethylthio-5'-*O*-(4,4'-dimethoxytrityl)-2'-deoxyadenosine (A8).** Compound **A7** (240 mg, 0.489 mmol) was coevaporated three times with dry pyridine (5 mL) and then dissolved in dry pyridine (2.5 mL). 4-(*N*,*N*-Dimethylamino)pyridine (8 mg, 0.049 mmol) was added. The mixture was treated with 4,4'-dimethoxytritylchloride (185 mg, 0.547 mmol; added in two portions over a period of one hour). Stirring of the yellow solution was continued for 14 h. Methanol (1 mL) was added before the solvents were evaporated, and the remaining residue coevaporated with methanol once more. The yellow foam was dissolved in dichloromethane, washed with aqueous 5% citric acid and saturated aqueous sodium bicarbonate solution, dried over sodium sulfate, and evaporated to give a white foam. The crude product was purified by column chromatography on $SiO_2$ (1.0–2.5% $CH_3OH$ in dichloromethane v/v) yielding **A8** as white foam (336 mg, 0.424 mmol, 87%). TLC (6% $CH_3OH$ in dichloromethane v/v) $R_f$ = 0.38. $^1H$ NMR (300 MHz, $d_6$-DMSO): $\delta$ 0.91 (m, 6H, $N(CH_2CH_2CH_2CH_3)_2$); 1.31, 1.32 (m, 4H, $N(CH_2CH_2CH_2CH_3)_2$); 1.58, 1.60 (m, 4H, $N(CH_2CH_2CH_2CH_3)_2$); 3.34 (m, 2H, $H_2C(5')$); 3.44 (t, *J* = 6.68 Hz, 2H, $N(CH_2CH_2CH_2CH_3)_2$); 3.59 (t, *J* = 7.07 Hz, 2H, $N(CH_2CH_2CH_2CH_3)_2$); 3.723 (s, 6H, 2x O-$CH_3$); 4.02 (m, 1H, H-C(4')); 4.49 (triplettoid, 1H, H-C(3')); 5.01 (triplettoid, 1H, H-C(2')); 6.35 (d, *J* = 8.52 Hz, 1H, H-C(1')); 6.55 (d, *J* = 4.71, 1H, HO-C(3')); 6.85 (triplettoid, 4H, H-C(ar)); 7.27, 7.29 (m, 6H, H-C(ar)); 7.40 (m, 3H, 2x H-C(ar), H-C(4ar)); 8.25 (s, 1H, H-C(8)); 8.44 (s, 1H, H-C(2)); 8.92 (s, 1H, H-C=N-C(6)) ppm. $^{13}C$ NMR (75 MHz, $d_6$-DMSO): $\delta$ 13.05, 13.69 (2x $N(CH_2CH_2CH_2CH_3)_2$); 19.11, 19.63 (2x $N(CH_2CH_2CH_2CH_3)_2$); 28.66, 30.43 (2x $N(CH_2CH_2CH_2CH_3)_2$); 44.49, 50.94 ($N(CH_2CH_2CH_2CH_3)_2$); 48.77 (C(2')); 54.97 (2x O-$CH_3$); 63.47 (C(5')); 72.22 (C(3')); 85.72 (C(4')); 88.50 (C(1')); 113.11–135.39 (C(ar)); 130.46 (q, *J* = 306.6 Hz, $SCF_3$); 141.95 (C(2)); 151.82 (C(8)) ppm. $^{19}F$ NMR (565 MHz, $d_6$-DMSO): $\delta$ –38.98 ppm. ESI-MS (m/z): $[M+H]^+$ calcd for $C_{41}H_{48}F_3N_6O_5S$ 793.38, found 793.33.

$^1H$ NMR (300 MHz, $d_6$-DMSO) compound **A8**

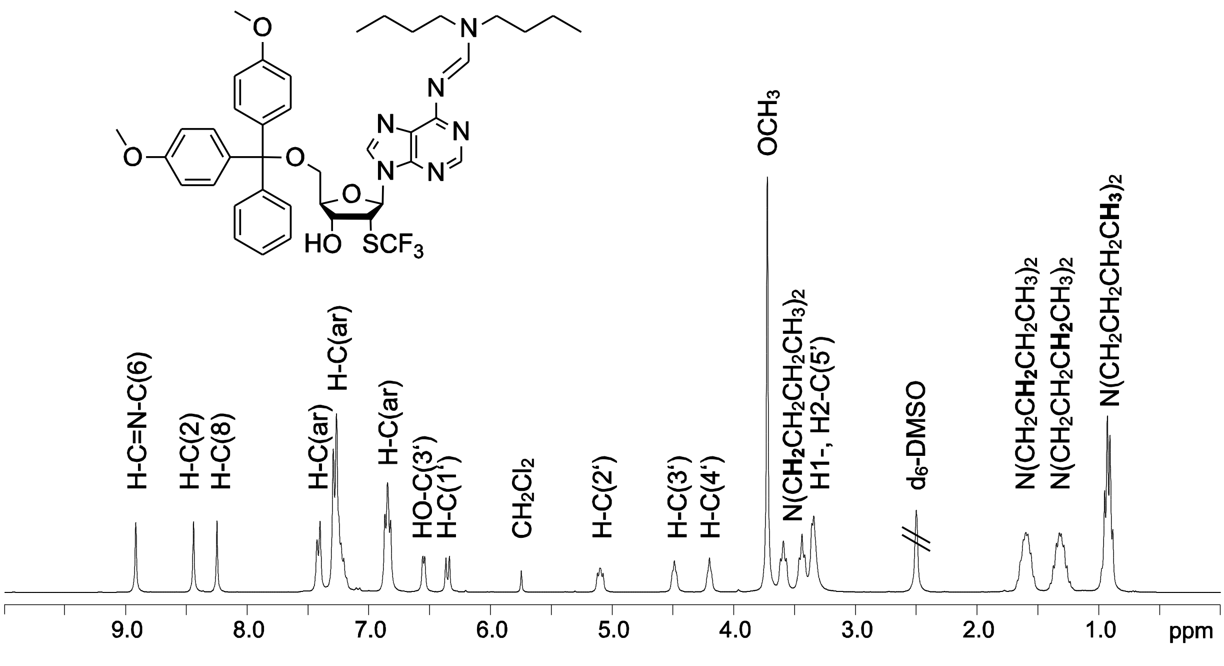

$^{13}$C NMR (75 MHz, $d_6$-DMSO) compound **A8**

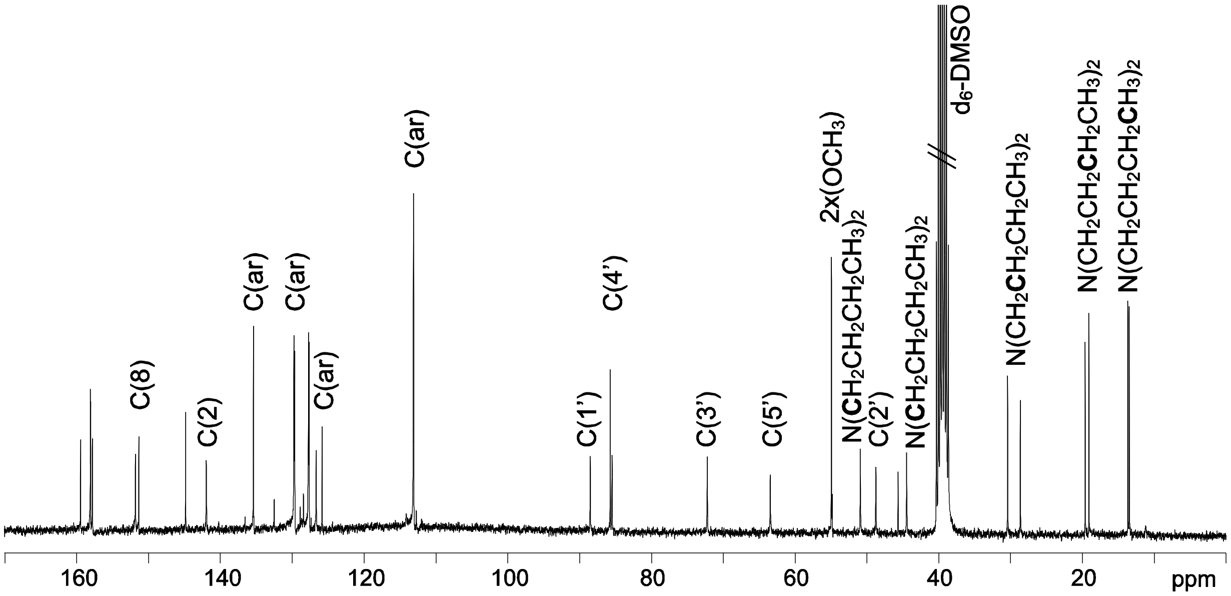

**$N^6$-[(Di-*n*-butylamino)methylene]-2'-trifluoromethylthio-5'-*O*-(4,4'-dimethoxytrityl)-2'-deoxyadenosine 3'-O-(2-cyanoethyl *N*,*N*-diisopropylphosphoramidite) (A9)**. Compound **A8** (243 mg, 0.306 mmol) was dissolved in 2 mL dichloromethane, N,N-ethyldimethylamine (330 µL, 3.05 mmol) was added and the solution was stirred for 0.5 hours. The mixture was treated with 2-cyanoethyl-*N*,*N*-diisopropylchlorophosphoramidite (109 mg, 0.461 mmol) for 3.5 hours. After that time the solvents were removed and the crude product purified by column chromatography on $SiO_2$ (5/5–6/4 ethylacetate/hexane v/v) yielding **A9** as white foam (222 mg, 73%). TLC (5/5 ethylacetate/hexane v/v) $R_f$ = 0.38. $^1H$ NMR (300 MHz, $CDCl_3$): $\delta$ 0.92 (m, 6H, 2x $N(CH_2CH_2CH_2CH_3)_2$); 1.20, 1.22 (m, 6H, 2x $CH(CH_3)_2$); 1.35 (m, 4H, 2x $N(CH_2CH_2CH_2CH_3)_2$); 1.61 (m, 4H, 2x $N(CH_2CH_2CH_2CH_3)_2$); 2.34, 2.58 (t, *J* = 6.24, 2H, 2x $CH(CH_3)_2$); 3.37 (m, 3H, H1-C(5'), $N(CH_2CH_2CH_2CH_3)_2$); 3.55–3.69 (m, 6H, $OCH_2CH_2CN$, $N(CH_2CH_2CH_2CH_3)_2$, 2x $CH(CH_3)_2$); 3.75 (s, 6H, 2x $OCH_3$); 3.89 (m, $OCH_2CH_2CN$); 4.09 (m, 2x $CH(CH_3)_2$); 4.38, 4.43 (s, 1H, H-C(4')); 4.69, 4.82 (m, 1H, H-C(3')); 5.00, 5.01 (m, 1H, H-C(2')); 6.22, 6.27 (2x d, *J* = 9.00, 8.88 Hz, 1H, H-C(1')); 6.80 (m, 4H, H-C(ar)); 7.31 (m, 6H, H-C(ar)); 7.42 (m, 3H, H-C(ar)); 7.99, 8.01 (s, 1H, H-C(8)); 8.37 (s, 1H, H-C(2)) ppm. $^{31}P$ NMR (121 MHz, $d_6$-$CDCl_3$): $\delta$ 151.19, 152.64 ppm. $^{19}F$ NMR (565 MHz, $CDCl_3$): $\delta$ –39.74, –39.82 ppm. ESI-MS (m/z): $[M+H]^+$ calcd for $C_{50}H_{65}F_3N_8O_6PS$ 993.44, found 993.38.

$^1H$-NMR (300 MHz, $CDCl_3$) compound **A9**

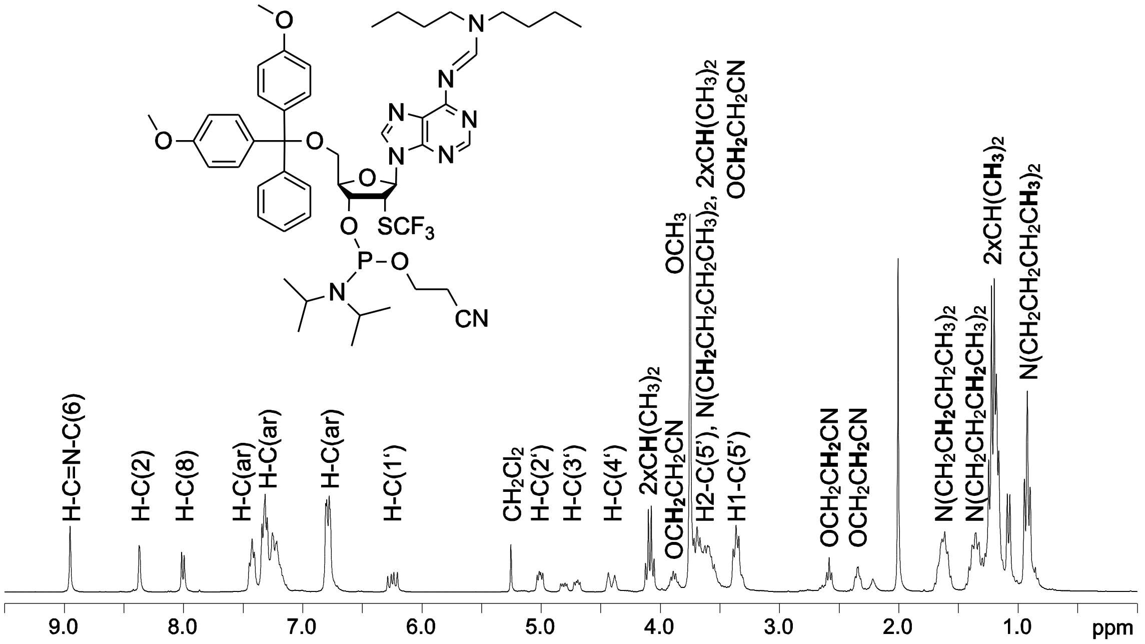


$^{13}C$-NMR (75 MHz, $CDCl_3$) compound **A9**

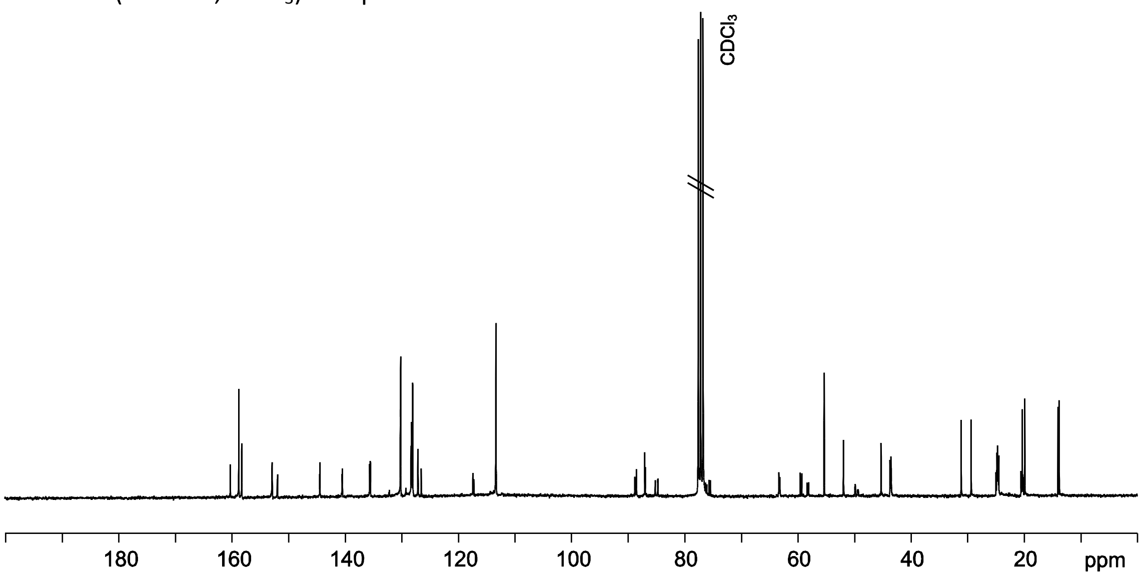

**2. NMR spectra of 2'-SCF$_3$ guanosine building block G7 and the precursors G1 to G6.**

***O$^6$-tert*-Butyl-*N,N*-bis(*tert*-butyloxycarbonyl)-3',5'-*O*-(1,1,3,3-tetra*iso*propylsiloxane-1,3-diyl)-arabinoguanosine (G1).**

$^1$H NMR (300 MHz, d$_6$-DMSO) of compound **G1**

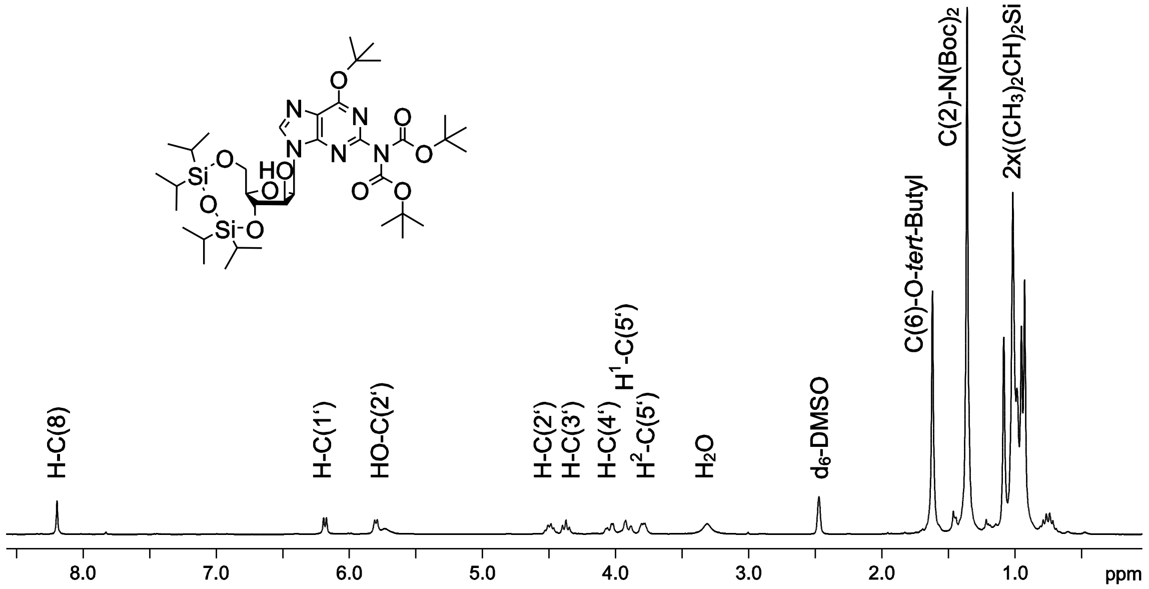


$^{13}$C NMR (75 MHz, d$_6$-DMSO) of compound **G1**

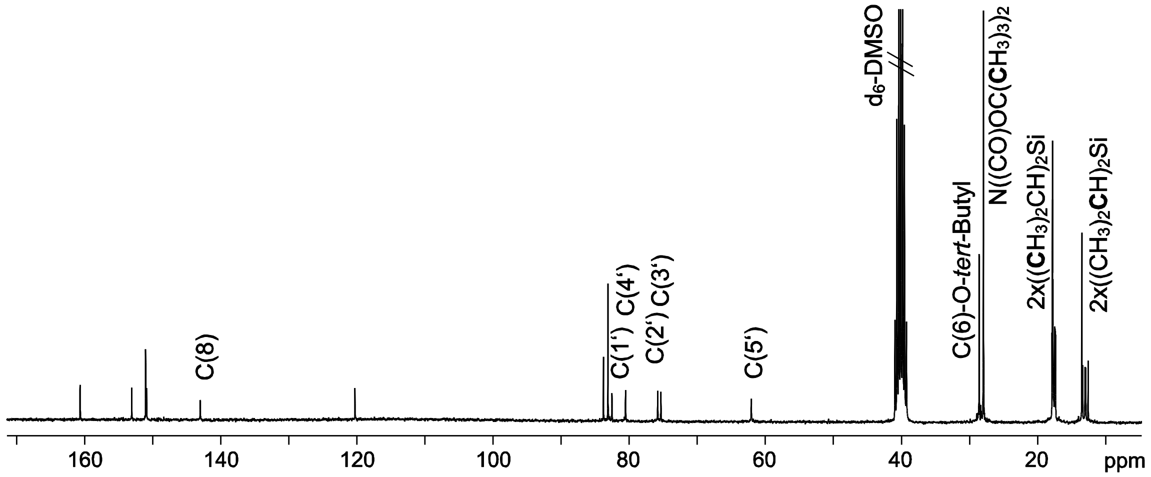

**$O^6$-*tert*-Butyl-*N,N*-bis(*tert*-butyloxycarbonyl)-3',5'-*O*-(1,1,3,3-tetra*iso*propylsiloxane-1,3-diyl)-2'-acetylthio-2'-deoxyguanosine (G2).**

$^1$H NMR (300 MHz, $CDCl_3$) of **G2**

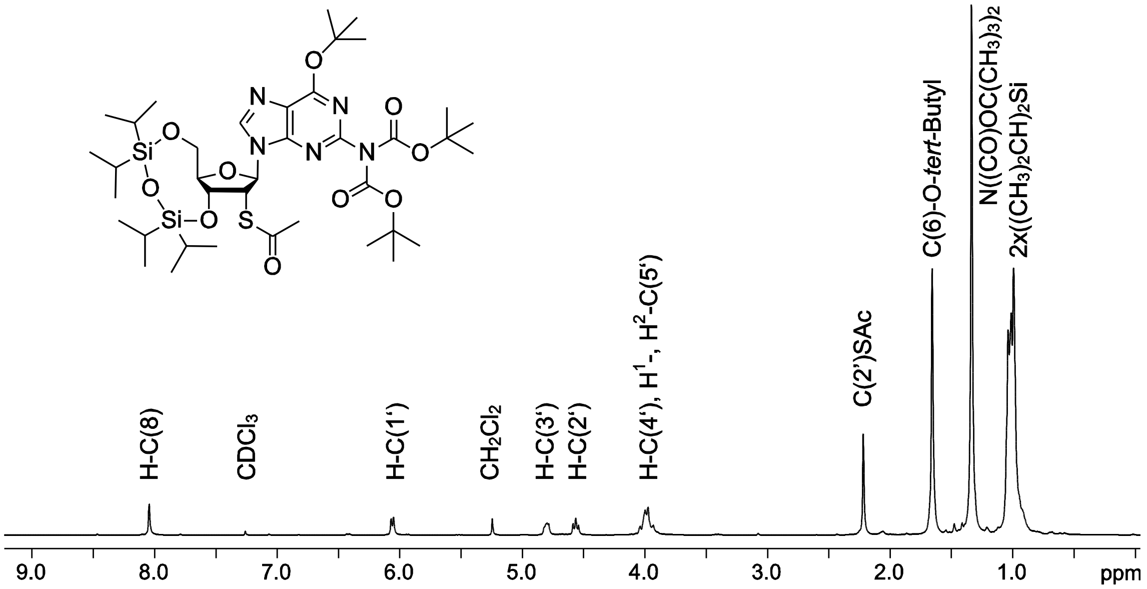


$^{13}$C NMR (75 MHz, $CDCl_3$) compound **G2**

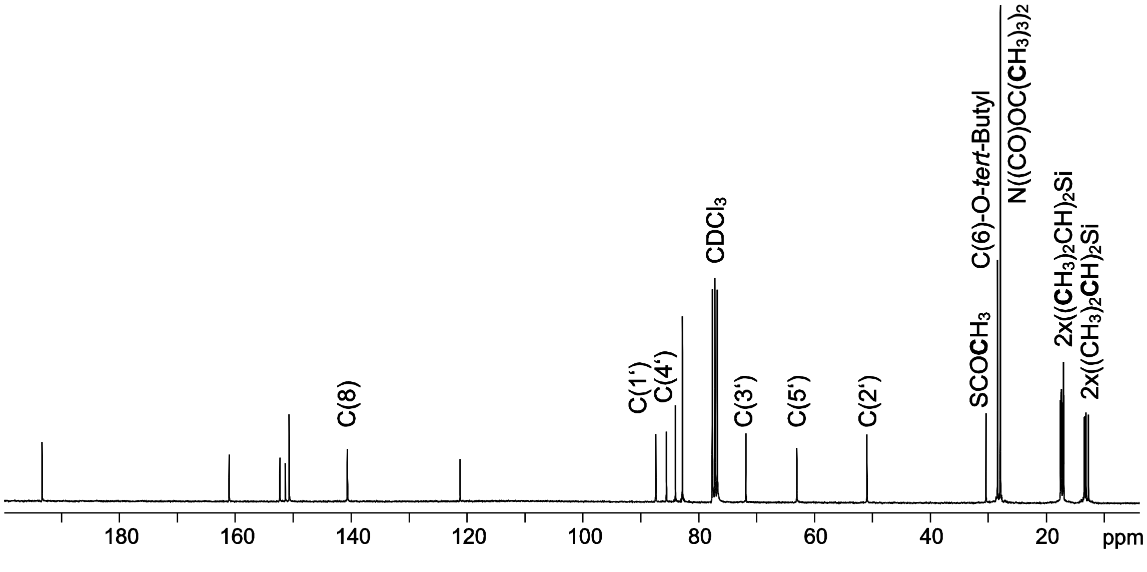

***O*$^6$-*tert*-Butyl-*N,N*-bis(*tert*-butyloxycarbonyl)-3′,5′-*O*-(1,1,3,3-tetra*iso*propylsiloxane-1,3-diyl)-2′-sulfanyl-2′-deoxyguanosine (G3)**.

$^1$H NMR (300 MHz, d$_6$-DMSO) of **G3**

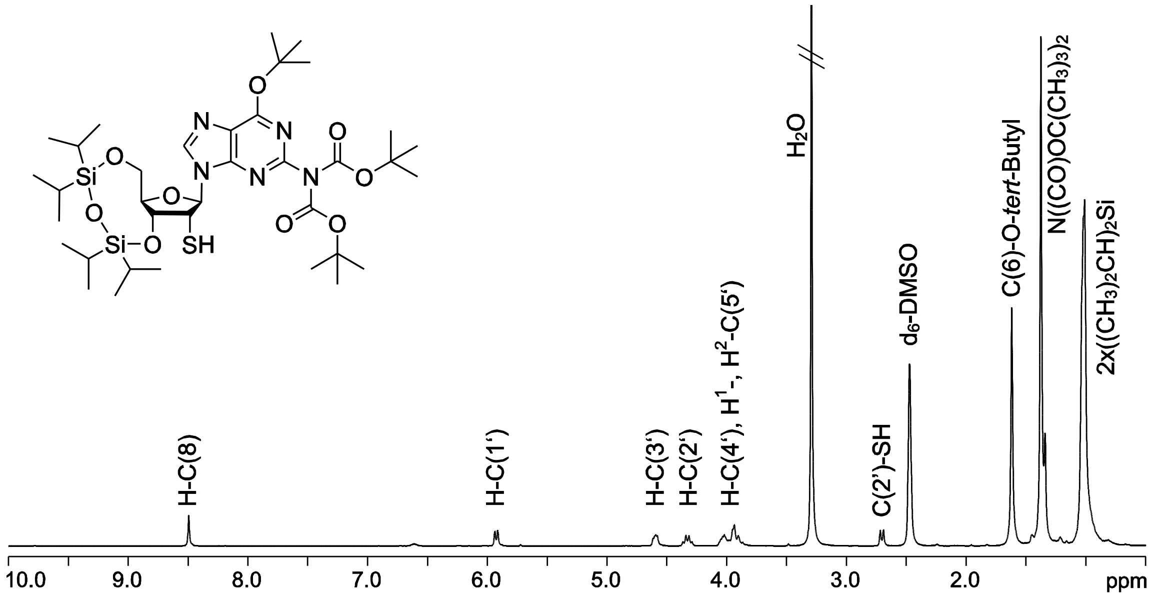


$^{13}$C NMR (75 MHz, d$_6$-DMSO) compound **G3**

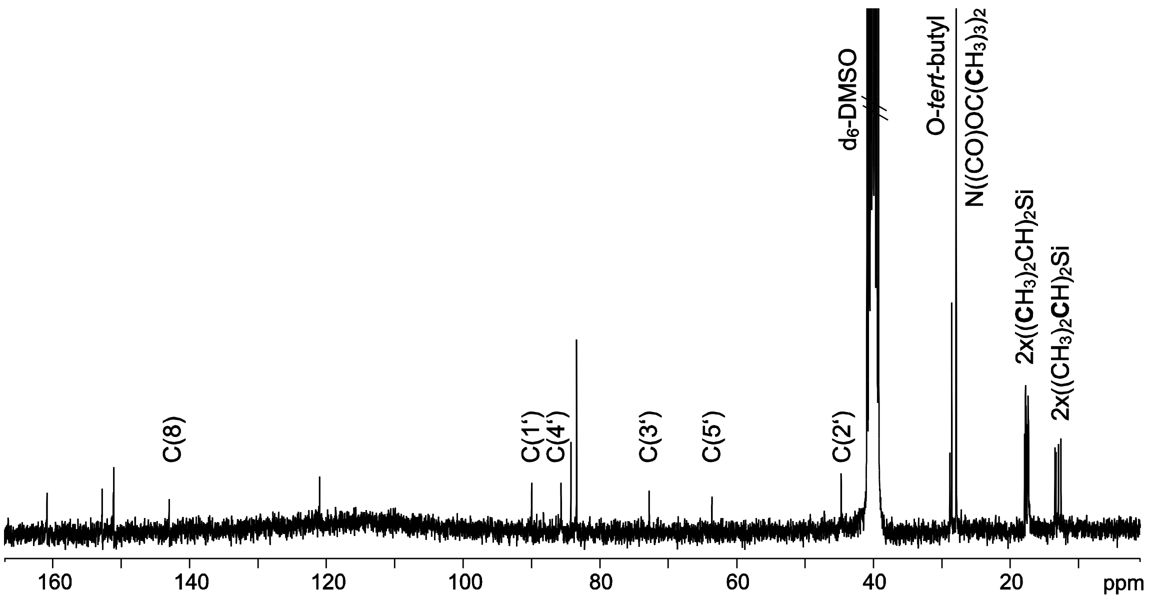

***O*⁶-tert*-Butyl-*N,N*-bis(*tert*-butyloxycarbonyl)-3′,5′-*O*-(1,1,3,3-tetra*iso*propylsiloxane-1,3-diyl)-2′-trifluoromethylthio-2′-deoxyguanosine (G4)**.

$^{1}$H NMR (300 MHz, $CDCl_3$) of **G4**

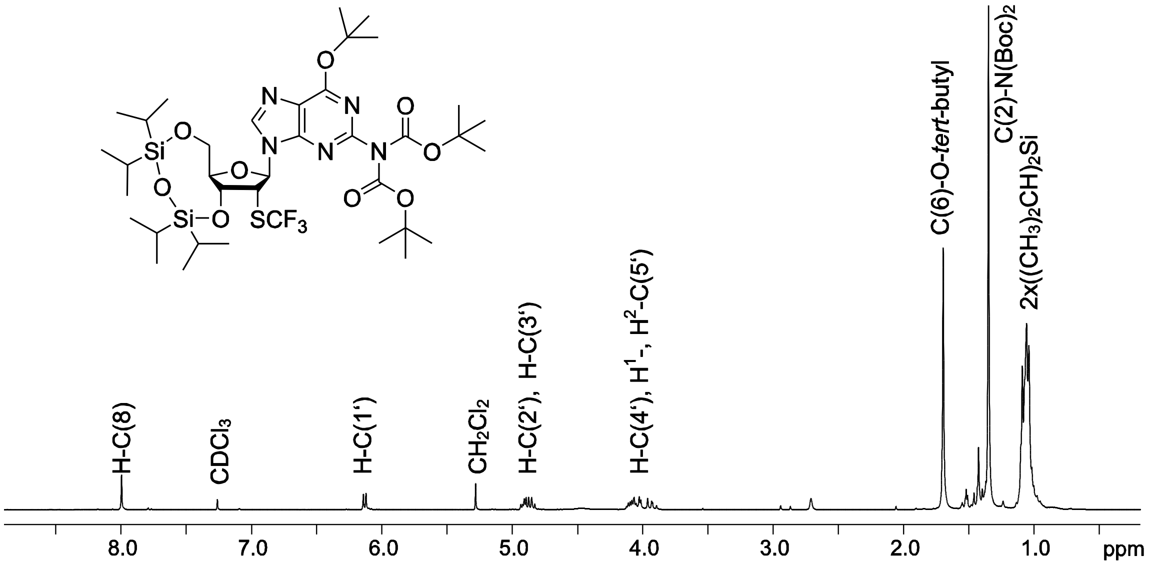


$^{13}$C NMR (75 MHz, $CDCl_3$) compound **G4**

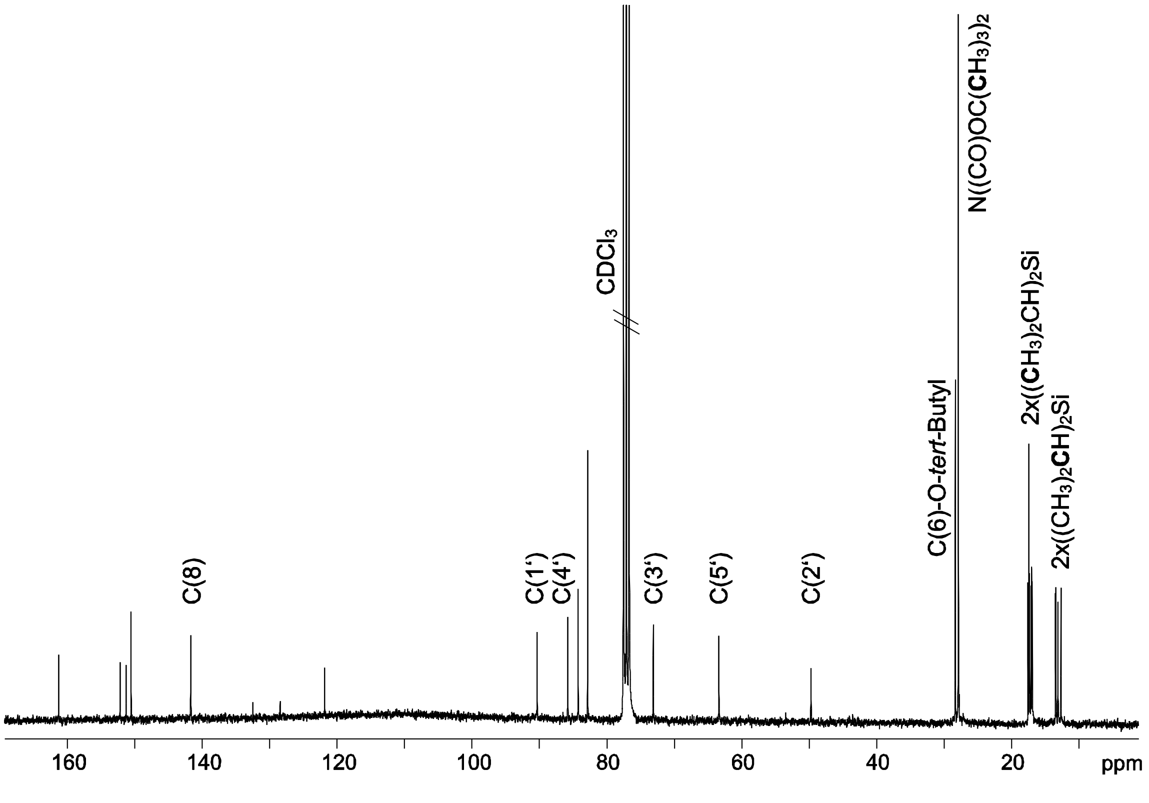

***O*[6]-*tert*-Butyl-*N,N*-bis(*tert*-butyloxycarbonyl)-2'-trifluoromethylthio-2'-deoxyguanosine (G5).**

$^{1}$H NMR (300 MHz, $d_6$-DMSO) of **G5**

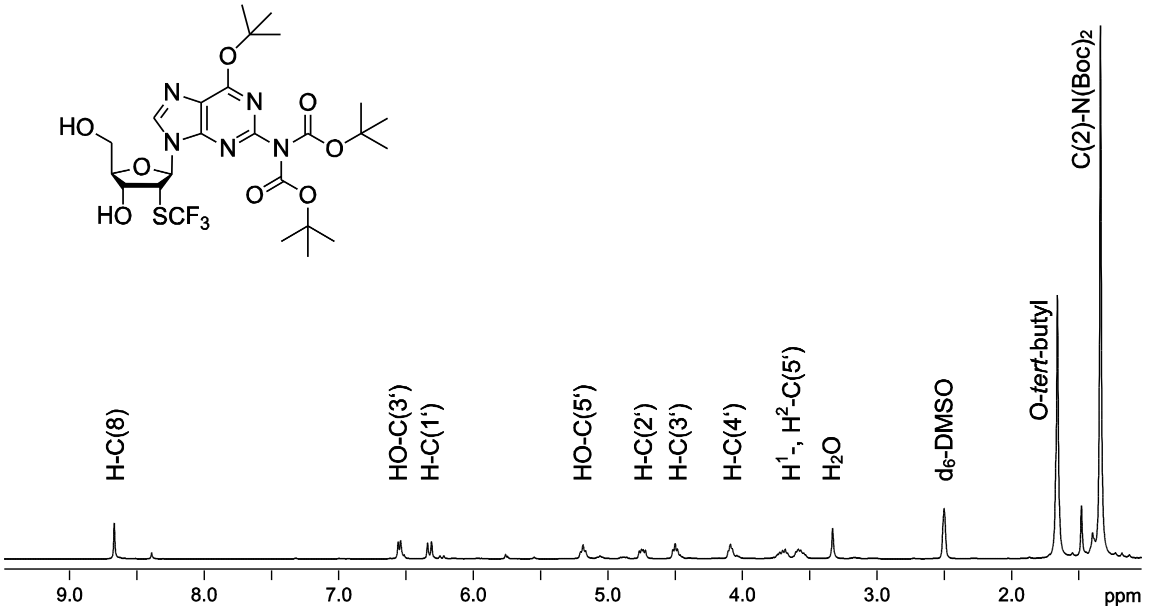


$^{13}$C NMR (75 MHz, $CDCl_3$) compound **G5**

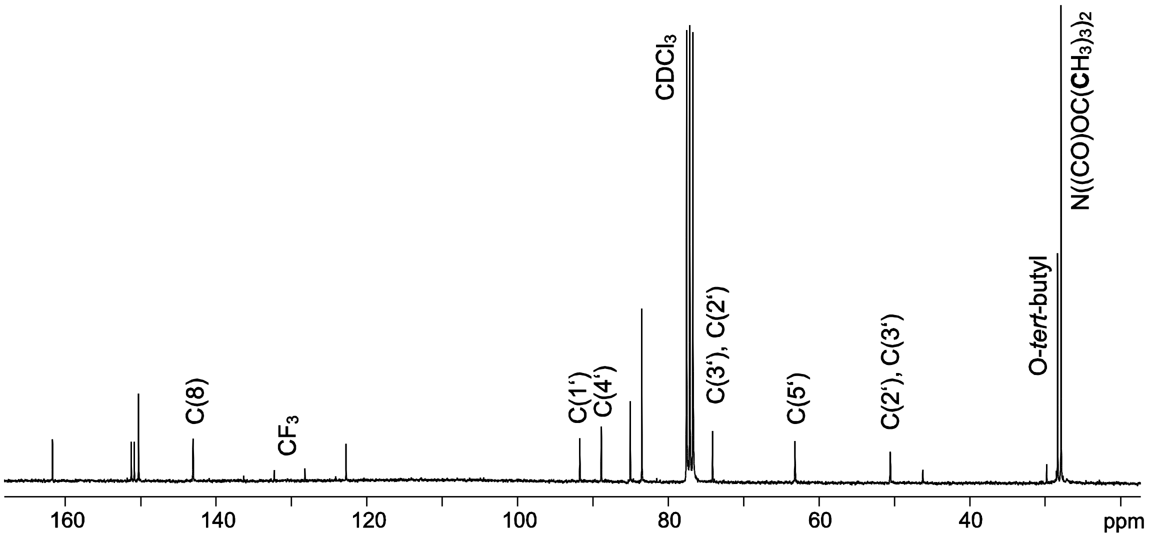

***N*[2]-[(Dimethylamino)methylene]-2’-trifluoromethylthio-5’-*O*-(4,4’-dimethoxytrityl)-2’-deoxyguanosine (G6).**

$^{1}H$ NMR (300 MHz, $CDCl_3$) of **G6**

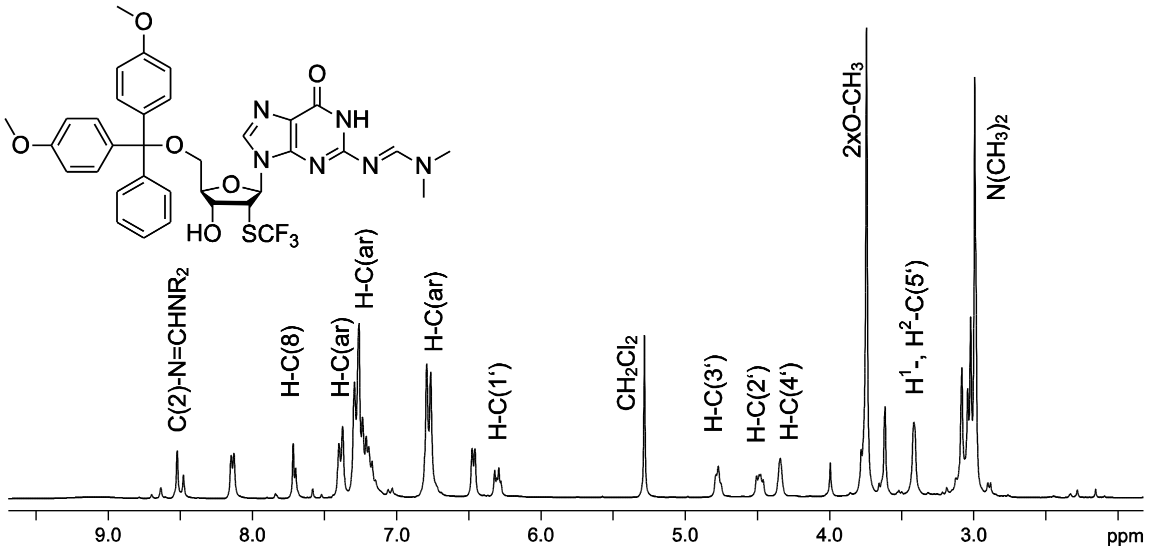


$^{13}C$ NMR (75 MHz, $CDCl_3$) compound **G6**

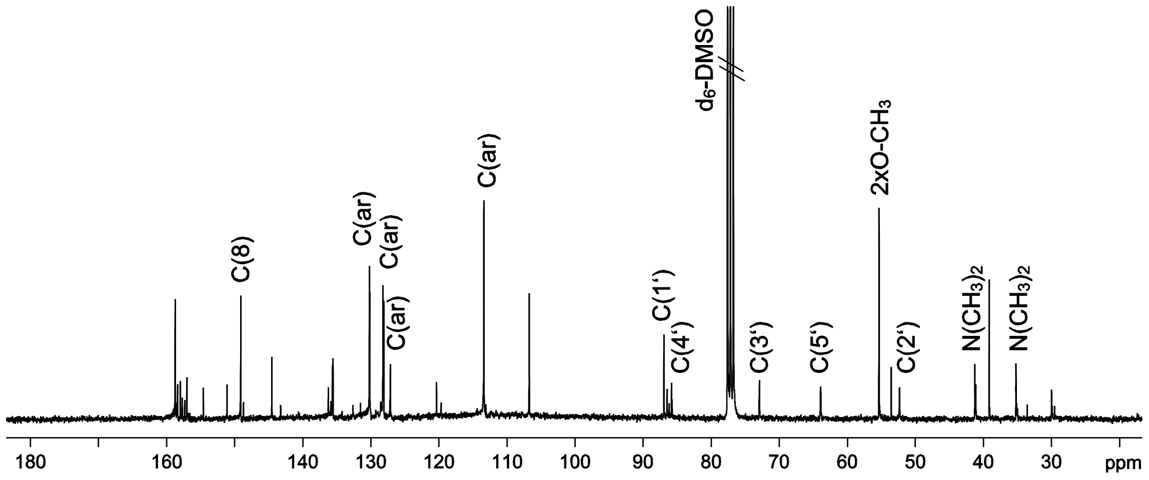

***N*²-[(Dimethylamino)methylene]-2′-trifluoromethylthio-5′-*O*-(4,4′-dimethoxytriphenylmethyl)-2′-deoxyguanosine 3′-*O*-((2-cyanoethyl) *N*,*N*-diisopropylphosphoramidite) (G7).**

$^{1}H$ NMR (300 MHz, $CDCl_3$) of **G7**

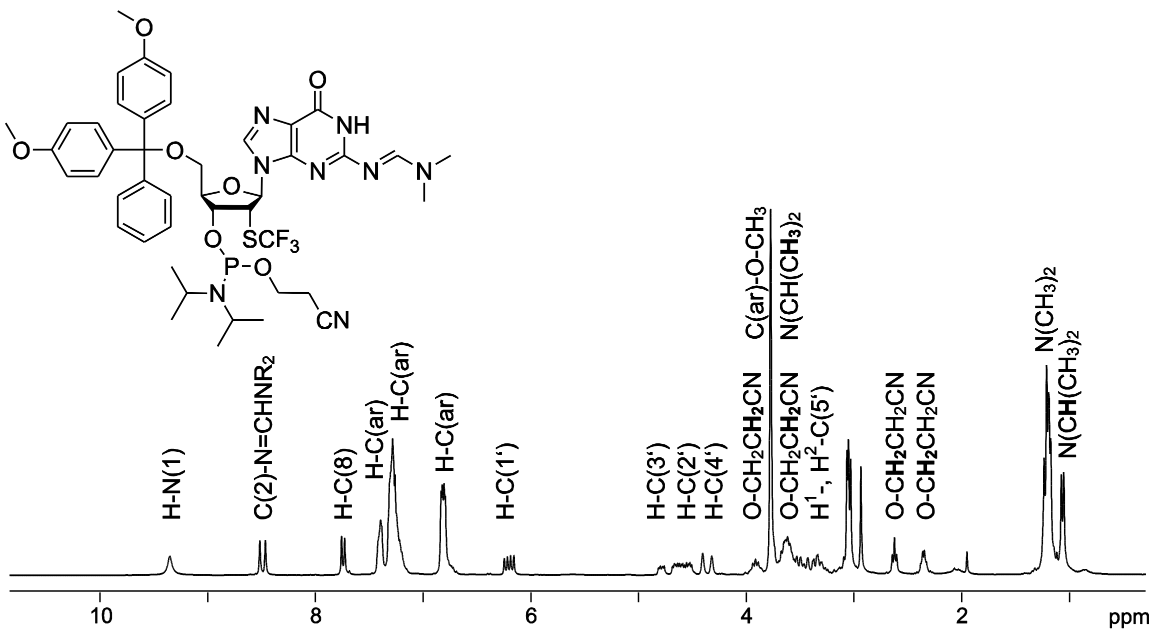


$^{13}C$ NMR (75 MHz, $CDCl_3$) compound **G7**

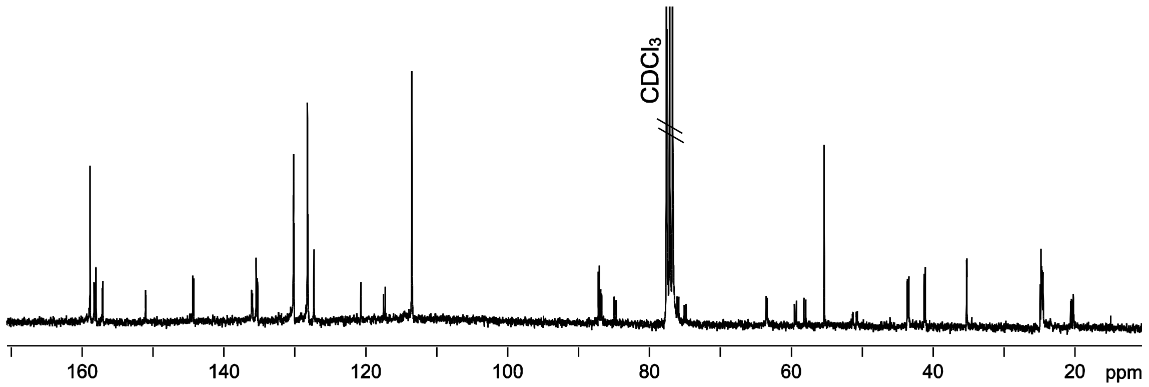

**3. Mass spectrometry of 2'-$SCF_3$ modified RNA**

All experiments were performed on a Finnigan LCQ Advantage MAX ion trap instrumentation connected to an Amersham Ettan micro LC system. RNA sequences were analyzed in the negative-ion mode with a potential of -4 kV applied to the spray needle. LC: Sample (200 pmol RNA dissolved in 30 µL of 20 mM EDTA solution; average injection volume: 30 µL); column (Waters XTerra® MS, C18 2.5 µm; 1.0 x 50 mm) at 21°C; flow rate: 30 µL/min; eluant A: 8.6 mM TEA, 100 mM 1,1,1,3,3,3-hexafluoroisopropanol in $H_2O$ (pH 8.0); eluant B: methanol; gradient: 0-100 % B in A within 30 min; UV-detection at 254 nm.

**4. $^{19}F$ and $^{1}H$ NMR experiments of 2'-$SCF_3$ modified RNA**

The RNA sample was lyophilized and dissolved in 25 mM sodium cacodylate buffer pH 7.0 (450 µL) and $D_2O$ (50 µL) was added to a total volume of 500 µL. RNA concentrations were as indicated in the corresponding figure captions. All samples were heated to 90 °C for 1 min, then rapidly cooled in an ice bath and equilibrated to room temperature for 15 min before measurements. The $^{1}H$ 1D-NMR spectra were acquired using a double-pulsed field gradient spin-echo pulse sequence. $^{19}F$ NMR spectra were recorded at a frequency of 564.6 MHz on a Bruker Avance II+ 600 MHz spectrometer equipped with a 5 mm QNP probe with Z-gradient ($^{2}H/^{1}H/^{19}F$). Typical experimental parameters were chosen as follows: spectral width 20 ppm, $^{19}F$ 90°-pulse 13 µs, acquisition time 1.2 s, relaxation delay 1.5 to 2.0 s. Prior to Fourier transformation all time domain data was processed with an exponential window function using a line broadening factor of 2-4 Hz. $^{19}F$ resonances are reported relative to external $CCl_3F$.

**5. UV-melting profile experiments and analysis**

Absorbance versus temperature profiles were recorded at 250, 260, 270, and 280 nm on a Cary-1 spectrophotometer equipped with a Peltier temperature control device. RNAs were measured at various concentrations ranging from ~1 to 60 µM in buffer solutions of 10 mM $Na_2HPO_4$, pH 7.0, containing 150 mM NaCl. Data were collected after a complete cooling and heating cycle at a rate of 0.7°C $min^{-1}$. Melting transitions were reversible and essentially the same with respect to the four different wavelengths. For sample preparation, oligonucleotides were lyophylized to dryness, dissolved in the corresponding buffer from stock solutions and subsequently degassed. A layer of silicon oil was placed on the surface of the solution. $\Delta H^{vH}$ and $\Delta S^{vH}$ values for biomolecular melting transitions were obtained from plots of $T_m^{-1}$ versus (ln c) plots where $\Delta H^{vH}$ and $\Delta S^{vH}$ are extracted from the slope and intercept of linear fits to the data. For monomolecular transitions, $\Delta H^{vH}$ and $\Delta S^{vH}$ were obtained from a two-state van't Hoff analysis by fitting the shape of the individual α versus temperature curve according to the literature (Marky, L. A., Breslauer, K. J. Calculating Thermodynamic Data for Transitions of Any Molecularity from Equilibrium Melting Curves. *Biopolymers* 1987, *26* (9), 1601–1620; Xia, T., McDowell, J. A., Turner, D. H. Thermodynamics of Nonsymmetric Tandem Mismatches Adjacent to G·C Base Pairs in RNA. *Biochemistry* 1997, *36* (41), 12486–12497.

**Table S1**. Selection of 2'-$SCF_3$ modified RNAs synthesized in the course of this study.

| RNA sequence (5'-3') | nt | Isolated total amount [nmol] | $M_r$ calc [amu] | $M_r$ found [amu][1] |
|---|---|---|---|---|
| UU$A_{2'SCF3}$ GCG | 6 | 505 | 1959.24 | 1959.02 |
| UU$A_{2'SCF3}$ GCG | 6 | 393 | 1959.25 | 1958.64 |
| UU$G_{2'SCF3}$ UUU | 6 | 778 | 1898.16 | 1897.83 |
| GGU CG$A_{2'SCF3}$ CC | 8 | 70 | 2608.63 | 2608.69 |
| GGU C$G_{2'SCF3}$A CC | 8 | 286 | 2608.65 | 2608.55 |
| CGG AGU $G_{2'SCF3}$AU CCG | 12 | 55 | 3934.45 | 3934.26 |
| GAA $G_{2'SCF3}$GG CAA CCU UCG | 15 | 157 | 4898.05 | 4898.13 |
| GA$A_{2'SCF3}$ GGG CAA CCU UCG | 15 | 169 | 4898.05 | 4897.69 |
| GGU CUC UGC CAA UA $A_{2'SCF3}$ GAC AUU | 21 | 94 | 6741.12 | 6740.82 |
| GGU CUC U $G_{2'SCF3}$C CAA UAA GAC AUU | 21 | 87 | 6741.12 | 6741.52 |
| UGU CUU AUU $G_{2'SCF3}$GC AGA GAC CUG | 21 | 98 | 6774.11 | 6774.52 |
| U$G_{2'SCF3}$U CUU AUU GGC $A_{2'SCF3}$GA GAC CUG | 21 | 135 | 6858.17 | 6858.25 |
| U$G_{2'SCF3}$U CUU AUU GGC AGA GAC CUG | 21 | 94 | 6774.11 | 6774.55 |
| UGC UCC UAG UAC GAG $A_{2'SCF3}$GG ACC GGA GUG | 27 | 152 | 8811.39 | 8811.88 |
| UGC UCC UAG UAC GAG AGG ACC GG$A_{2'SCF3}$ GUG | 27 | 165 | 8811.39 | 8811.41 |
| UGC UCC U$G_{2'SCF3}$G UAC GAG AGG ACC GGA GUG | 27 | 73 | 8811.39 | 8811.23 |
| UGC UCC UA$G_{2'SCF3}$ UAC GAG AGG ACC GGA GUG | 27 | 159 | 8811.39 | 8811.75 |
| UGC UCC UAG UAC GAG AGG ACC $G_{2'SCF3}$GA GUG | 27 | 191 | 8811.39 | 8811.76 |
| CUG GGU CGC AGU AAC CCC AGU UAA CAA AAC AAG $G_{2'SCF3}$G | 35 | 101 | 11356.98 | 11357.23 |

[1] determined by LC-ESI mass spectrometry

**Table S2**. Selection of 2’-$SCF_3$ modified siRNAs investigated in this study.

| Synonym | siRNA duplex | Mr calcd [g/mol] | Mr found [g/mol] |
|---|---|---|---|
| SIR3 unmod | 5'- G G U C U C U G C C A A U A A G A C A U U -3' | 6657.06 | 6657.39 |
| | 3'- G U C C A G A G A C G G U U A U U C U G U -5' | 6690.05 | 6690.39 |
| SIR3 2'-SCF3-A15 s | 5'- G G U C U C U G C C A A U A A G A C A U U -3' | 6741.12 | 6740.82 |
| | 3'- G U C C A G A G A C G G U U A U U C U G U -5' | 6690.05 | 6690.39 |
| SIR3 2'-SCF3-G8 s | 5'- G G U C U C U G C C A A U A A G A C A U U -3' | 6741.12 | 6741.52 |
| | 3'- G U C C A G A G A C G G U U A U U C U G U -5' | 6690.05 | 6690.39 |
| SIR3 2'-SCF3-G10 as | 5'- G G U C U C U G C C A A U A A G A C A U U -3' | 6657.06 | 6657.39 |
| | 3'- G U C C A G A G A C G G U U A U U C U G U -5' | 6774.11 | 6774.52 |
| SIR3 2'-SCF3-G2 as | 5'- G G U C U C U G C C A A U A A G A C A U U -3' | 6657.06 | 6657.39 |
| | 3'- G U C C A G A G A C G G U U A U U C U G U -5' | 6774.11 | 6774.55 |
| SIR3 2'-SCF3-G2/A13 as | 5'- G G U C U C U G C C A A U A A G A C A U U -3' | 6657.06 | 6657.39 |
| | 3'- G U C C A G A G A C G G U U A U U C U G U -5' | 6858.17 | 6858.25 |
| SIR3 random | 5'- U C U G G G U C U A A G C C A A A C A U T -3' | 6674.03 | 6674.39 |
| | 3'- dG U A G A C C C A G A U U C G G U U U G U -5' | 6655.06 | 6655.01 |

A=2'-SCF3 Adenosine, G=2'-SCF3 Guanosine

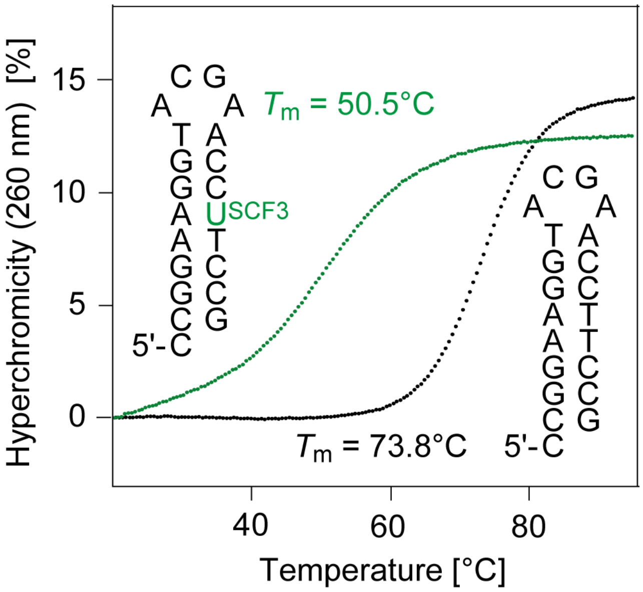


**Supporting Figure S1**. Thermal stabilities of a 2’-$SCF_3$ uridine modified DNA hairpin. Overlay of UV melting profiles of modified and unmodified DNA hairpins. Conditions: $c_{DNA}$ = 4 µM; 10 mM $Na_2HPO_4$, 150 mM NaCl, pH 7.0.

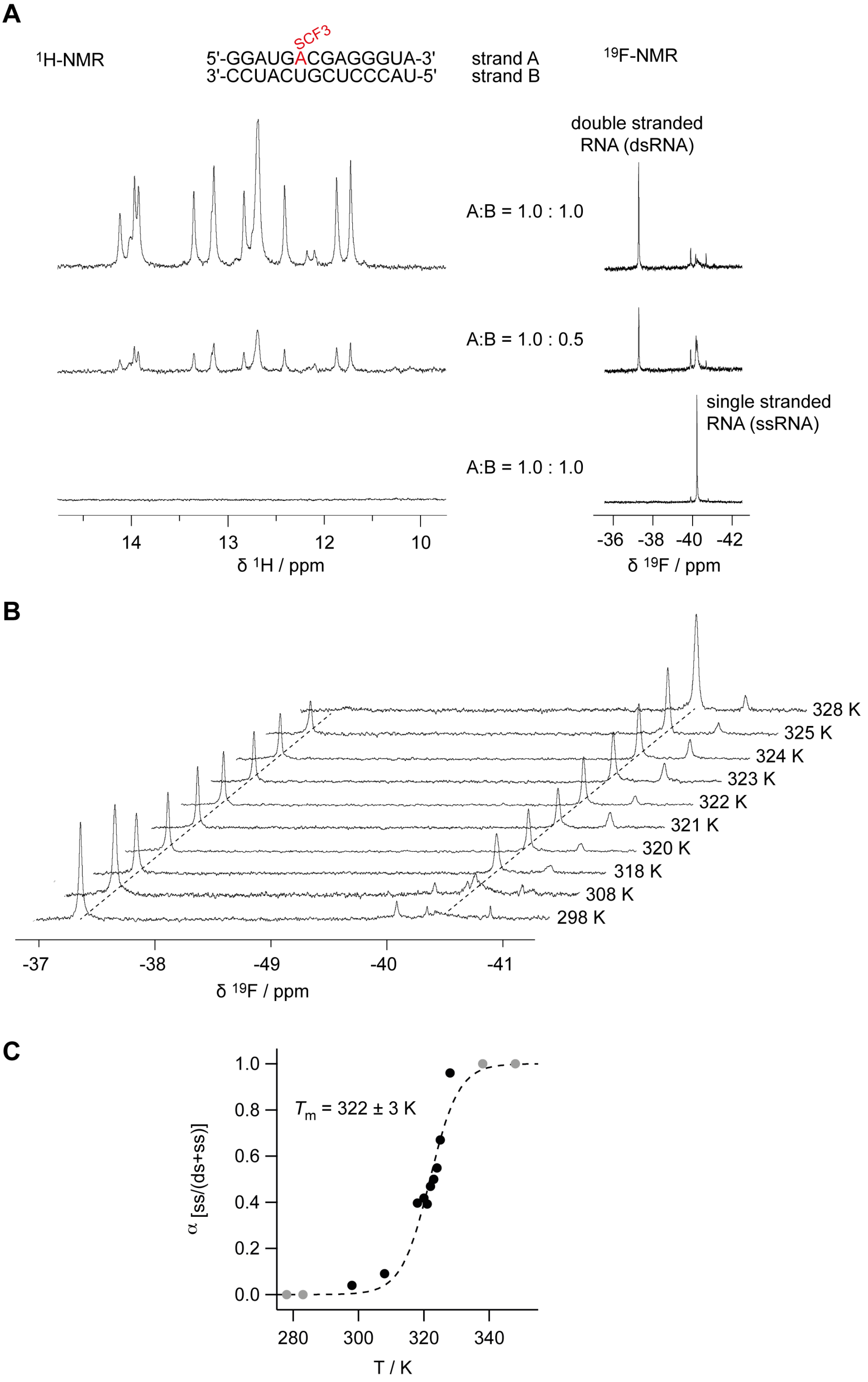


**Supporting Figure S2**. Duplex formation of 2'-$SCF_3$ adenosine modified RNA. **A**) Titration experiment followed by 1D $^{1}H$ and $^{19}F$ NMR spectroscopy; conditions: $c_{RNA}$ = 0.23 mM, 50 mM $Na_2HPO_4$, pH 6.5, 298 K; **B**) $^{19}F$ NMR spectra at different temperatures; **C**) Melting profile and estimated $T_m$ value derived from $\alpha/T$ graph of ∂F.

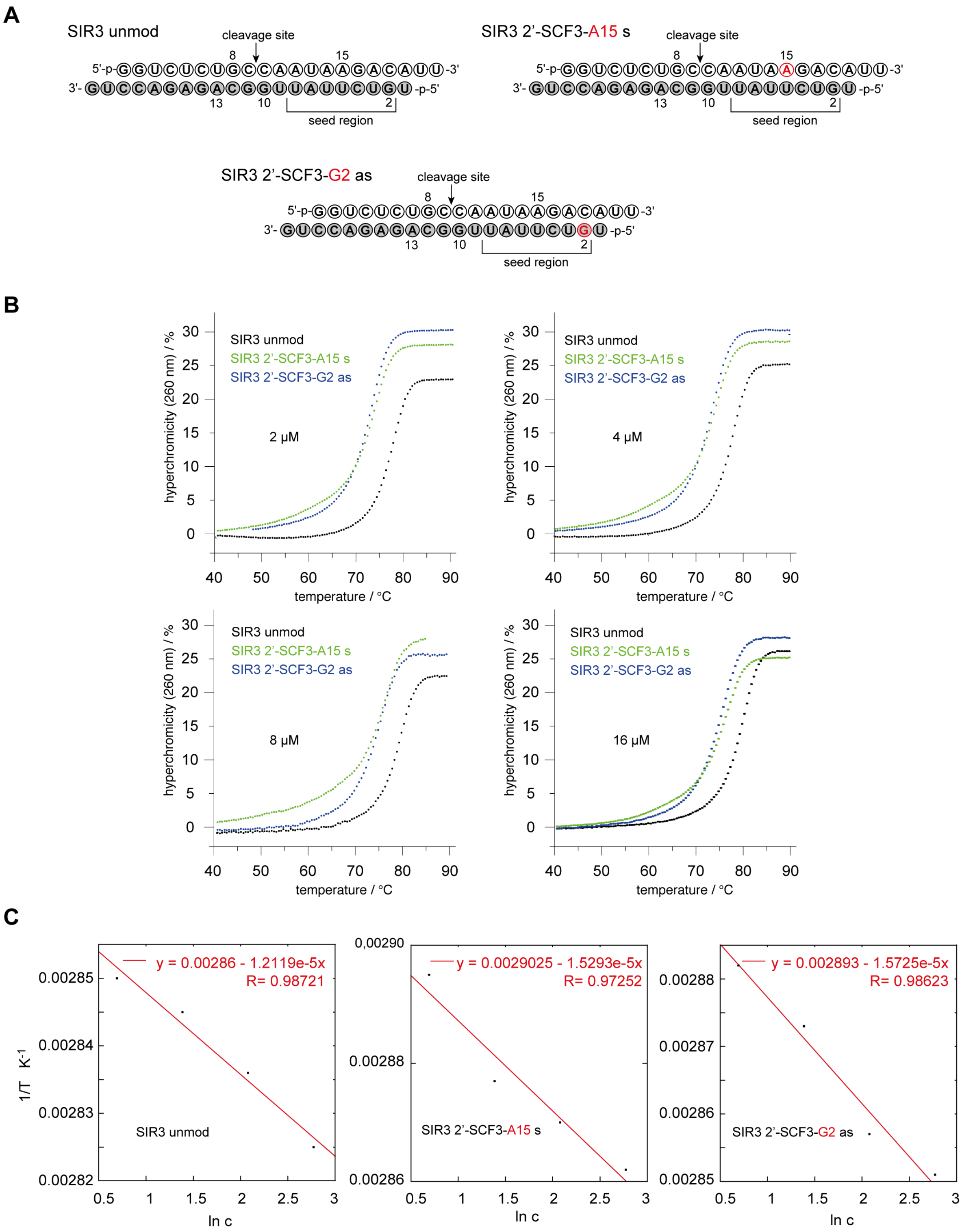


D

| siRNA | ΔH° | ΔS° | ΔG° |
|---|---|---|---|
| SIR3 unmod | -164.0 | -0.469 | -24.2 |
| SIR3 2'-SCF3-A15 s | -129.9 | -0.377 | -17.5 |
| SIR3 2'-SCF3-G2 as | -126.4 | -0.366 | -17.4 |

**Supporting Figure S3**. Thermodynamic analysis of siRNA duplexes with single 2'-$SCF_3$ modifications. **A**) RNA sequences; position of 2'-$SCF_3$ indicated in red. **B**) UV melting profiles of the three siRNA duplexes; concentrations as indicated. **C**) 1/T *versus* ln *c* graphs; **D**) Thermodynamic parameters derived from 1/T *versus* ln *c* graphs. Conditions: $c_{RNA}$ as indicated; 10 mM $Na_2HPO_4$, 150 mM NaCl, pH 7.0.
